# A high-throughput platform for detailed lipidomic analysis of a range of mouse and human tissues

**DOI:** 10.1007/s00216-020-02511-0

**Published:** 2020-03-07

**Authors:** Samuel Furse, Denise S. Fernandez-Twinn, Benjamin Jenkins, Claire L. Meek, Huw E. L. Williams, Gordon C. S. Smith, D. Stephen Charnock-Jones, Susan E. Ozanne, Albert Koulman

**Affiliations:** 1 Metabolic Research Laboratories and MRC Metabolic Diseases Unit, Wellcome Trust-MRC Institute of Metabolic Science, University of Cambridge, Box 289, Cambridge Biomedical Campus, Hills Road, Cambridge, CB2 0QQ UK; 2 Core Metabolomics and Lipidomics Laboratory, Wellcome Trust-MRC Institute of Metabolic Science, University of Cambridge,, Box 289, Cambridge Biomedical Campus, Cambridge, CB2 0QQ UK; 3 Department of Clinical Biochemistry/Wolfson Diabetes & Endocrine Clinic, Cambridge University Hospitals NHS Foundation Trust, Cambridge, CB2 0QQ UK; 4 Centre for Biomolecular Sciences, School of Chemistry, University of Nottingham, University Park, Nottingham, NG7 2RD UK; 5 Department of Obstetrics and Gynaecology, NIHR Cambridge Biomedical Research Centre, University of Cambridge, Cambridge, CB2 0SW UK; 6 Centre for Trophoblast Research, University of Cambridge, Cambridge, CB2 3EG UK

**Keywords:** Lipidomics, Lipid profiling, Mass spectrometry, 31P NMR, Metabolic disease, Mouse model, Human development

## Abstract

Lipidomics is of increasing interest in studies of biological systems. However, high-throughput data collection and processing remains non-trivial, making assessment of phenotypes difficult. We describe a platform for surveying the lipid fraction for a range of tissues. These techniques are demonstrated on a set of seven different tissues (serum, brain, heart, kidney, adipose, liver, and vastus lateralis muscle) from post-weaning mouse dams that were either obese (> 12 g fat mass) or lean (<5 g fat mass). This showed that the lipid metabolism in some tissues is affected more by obesity than others. Analysis of human serum (healthy non-pregnant women and pregnant women at 28 weeks’ gestation) showed that the abundance of several phospholipids differed between groups. Human placenta from mothers with high and low BMI showed that lean placentae contain less polyunsaturated lipid. This platform offers a way to map lipid metabolism with immediate application in metabolic research and elsewhere.

**Electronic supplementary material:**

The online version of this article (10.1007/s00216-020-02511-0) contains supplementary material, which is available to authorized users.

**Figure:**
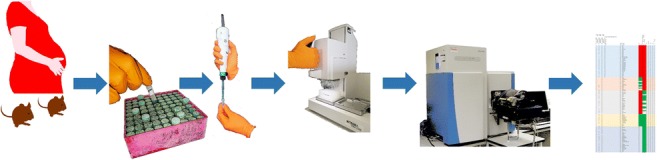
Graphical abstract

## Introduction

Detailed molecular surveys of the lipid fraction of biological samples (lipidomics) are of increasing interest in research of metabolism, development, and disease. They offer an insight into both energy storage, energy distribution, and structural changes at the membrane level. The power of lipidomic analysis is increasing further through ongoing development of techniques for characterizing the lipid fraction of biological samples [1–5] and has led to the development of a number of catalogues and databases detailing the known chemical and physical facets of known phospholipids [6–11].

There is mounting evidence that lipid metabolism has a crucial role in growth and metabolic disease in humans [12–16]. This has encouraged the development of methods required for lipid profiling. Such studies are typically based on plasma or serum samples from large numbers of human participants. With meta-data, this type of sample set can be used to give a useful snapshot of the metabolic or disease status of the participants. However, the interest has also led to questions that cannot be answered by determining the lipid composition of the circulating plasma or serum alone. Answering such questions requires an understanding of the lipid metabolism within and between organs, such as the liver and brain, across the life-course. This requires lipid profiling of more than one tissue, often at the same time, from the same individual animal and even longitudinally.

However, the liver and other such organs contain fibrous components that make the lipid fraction less accessible than it is in serum. This fibrosity makes extraction of lipids more difficult and measurements less consistent. Furthermore, whole-cell samples typically contain lipases that remain active ex vivo and can modify the lipids present, corrupting the final lipid profile acquired [17]. These factors make it yet more challenging to process tissue samples in a high-throughput manner, meaning that high-powered studies are less feasible.

This led us to develop a comprehensive pipeline that is amenable to investigation of lipid metabolism simultaneously across several tissues. We define pipeline as a series of methods that encompass all the steps necessary to acquire an interpretable lipid profile of a tissue sample. In this case, individual steps can be used in isolation or replace existing techniques in other pipelines or workflows.

The pipeline we developed was applied to investigate the lipid profile of two groups of mice (one lean and one obese) that represent a model of obesity with gestational diabetes [18–20]. Mouse brain, liver, heart, kidney, vastus lateralis muscle, and adipose tissue were run in the same sample batch with serum from the same individual animals. This was used to produce a set of lipid surveys that characterise the lipid metabolism in the two groups through their internal systems.

We used human serum samples from both pregnant and non-pregnant women taking part in an oral glucose tolerance test (OGTT) to explore the scope of this platform to uncover the shifts in lipid metabolism within tests of metabolic health, from a well-established sample type. Blood samples were taken from pregnant women at 28 weeks gestation and non-pregnant healthy control women (similar age and BMI) at fasting and 2 h after the glucose challenge. Lastly, we describe analysis of a set of human placentae from obese and lean women who participated in the Pregnancy Outcome Prediction (POP) study [21, 22].

A method for extracting the lipid and triglyceride fraction in a manner that is sympathetic to the chemical sensitivity of lipids is described and is followed by molecular profiling using both direct infusion mass spectrometry (DI-MS) and liquid chromatography with mass spectrometry (LC-MS) and phosphorus NMR (^31^P NMR). Methods for processing, basic analysis, and interpreting these data are then described. The pipeline is sufficiently flexible to allow for requirements of a variety of study designs and sizes. For example, we use ^31^P NMR with the expectation that in most high-powered studies, sufficient material for a sample will require pooling of samples from each phenotype.

The pipeline developed can be used on a particular sample type or as a platform for several sample types using high-throughput lipidomics, providing data on whole-body lipid profiling.

## Materials and methods

### Tissue and serum sample acquisition from mice

Mouse tissue samples were taken from post-weaning dams that were lean or obese. The latter represents a model of previous GDM [18–20]. Female C57BL/6J mice were randomly assigned to either a standard chow RM1 diet (approx. 7% mono/disaccharides, 3% fat, 50% polysaccharide, 15% protein [*w/w*], 10.74 kJ/g) or a highly palatable, energy-rich, semi-synthetic obesogenic diet (approx. 10% mono/disaccharides, 20% animal lard, 28% polysaccharide, 23% protein [*w/w*], 28.43 kJ/g) supplemented with sweetened condensed milk (Nestle, UK) (approx. 16% fat, 33% mono/disaccharides, 15% protein, 13.7 kJ/g) and fortified with the vitamin and mineral mixture AIN93G. All feeds were purchased from Special Dietary Services (Witham, UK). After 3 weeks fed *ad libitum*, females were mated with chow-fed males (first pregnancy). Dams remained on the same diets throughout gestation and lactation. The first litter was culled after they had been weaned. The first pregnancy ensured fertility and nurturing in the experimental mice. Seven days after weaning, body composition in the female dams was measured by time-domain nuclear magnetic resonance. As in previous work, the obese dams’ body composition was around 30–35% fat and that body weight did not exceed 35 g prior to mating for the second pregnancy [18]. The control (chow-fed) dams’ body composition was not permitted to exceed 10–12% prior to mating for the second pregnancy.

Samples of tissues from lean (< 5 g absolute fat mass) and obese (> 12 g absolute fat mass) post-weaning dams were used. Mice were culled by increasing the concentration of carbon dioxide, tissues dissected immediately, flash-frozen, and stored at − 80 °C. Blood was sampled by cardiac puncture and the serum obtained, snap-frozen, and stored at −80 °C.

### Tissue and serum sample acquisition from humans

Human serum samples came from pregnant (28 weeks’ gestation) and non-pregnant women and were collected at fasting (0 h) and 2 h postprandial as part of an oral glucose tolerance test. Participant data is shown in Electronic Supplementary Material (ESM) Table S1).

Villous human placenta samples were acquired from the Pregnancy Outcome Prediction Study (POPS) [22, 23]. All participants gave written informed consent. Samples from 79 placentae from live, singleton, vaginal births from mothers who were either lean (BMI 20–24, *n* = 39) or obese (BMI > 30, *n* = 40) were used (ESM Table S2).

#### Chemicals and reagents

Fine chemicals and HPLC-grade solvents were purchased from Sigma Aldrich-Merck plc (Gillingham, Dorset, UK) and used without further purification. Phospholipid, sterol, and triglyceride standards were purchased from Avanti Polar Lipids (Alabama, USA).

### Stock solutions

1. GCTU. Guanidine (6 M guanidinium chloride) and thiourea (1·5 M) were dissolved in deionised H_2_O together and stored at room temperature out of direct sunlight.
2. DMT. Dichloromethane (3 parts), methanol (1 part) and triethylammonium chloride (0·0005 parts, *i.e.* 500 mg/L) were mixed and stored at room temperature out of direct sunlight.
3. MS-mix. Propan-2-ol (2 parts) was mixed with methanol (1 part) and used to produce a solution of CH_3_COO.NH_4_ (7·5 mM).

### Tissue sample preparation for extraction of the lipid fraction

Samples of mouse brain, heart, vastus lateralis muscle, liver, kidney, and adipose tissue and human placenta were processed into pipettable solutions using GCTU and a hand-held homogeniser (TissueRuptor II, Qiagen, unless otherwise stated), see ESM Table S3. Fibrous tissues such as vastus lateralis benefitted from freeze-thawing. Serum samples were not treated with GCTU.

### Extraction of the lipid fraction

Solutions of homogenised organ preparations (volumes given in ESM Table S3; calculated to give ~1 μL lipid mixture per sample extraction) were injected into a well (96-well plate, Esslab Plate+™, 2·4 mL/well, glass-coated) followed by internal standards (150 μL, mixture of internal standards in methanol (see ESM Table S4), water (500 μL), and DMT (500 μL) using a 96-channel pipette. The mixture was agitated (96-channel pipette) before being centrifuged (3·2 k × *g*, 2 min). A portion of the organic solution (20 μL) was transferred to a high-throughput plate (384-well, glass-coated, Esslab Plate+™) before being dried (N_2 (g)_). When 4 × 96-well plates had been placed in the 384 well and the instrument was available, the dried films were re-dissolved (*tertiary-*butylmethyl ether, 20 μL/well, and MS-mix, 80 μL/well) and the plate was heat-sealed and queued immediately, with the first injection within 10 min.

Samples with a high concentration of triglycerides (TGs; *e.g.* adipose tissue) were treated as follows to concentrate the phospholipid fraction so it too could be profiled [24]: A second portion of the organic phase from the extraction (100 μL) of was transferred to a shallow plate (96-well, glass-coated) before being dried (N_2 (g)_), washed (hexane, 3×100 μL/well), and re-dissolved (DMT, 30 μL). The samples were transferred immediately to the high-throughput analytical plate as above and dried (N_2 (g)_).

### Direct infusion mass spectrometry

All samples were infused into an Exactive Orbitrap (Thermo, Hemel Hampstead, UK), using a TriVersa NanoMate (Advion, Ithaca US), for direct infusion mass spectrometry (DI-MS [16, 25]). Samples (15 μL ea.) were sprayed at 1·2 kV in the positive ion mode. The Exactive started acquiring data 20 s after sample aspiration began. The Exactive acquired data with a scan rate of 1 Hz (resulting in a mass resolution of 100,000 full width at half-maximum [fwhm] at 400 *m/z*). The Automatic Gain Control was set to 3,000,000 and the maximum ion injection time to 50 ms. After 72 s of acquisition in positive mode the NanoMate and the Exactive switched over to negative ionisation mode, decreasing the voltage to − 1·5 kV and the maximum ion injection time to 50 ms. The spray was maintained for another 66 s, after which the NanoMate and Exactive switched over to negative mode with in-source fragmentation (also known as collision-induced dissociation, CID; 70 eV) for a further 66 s. After this time, the spray was stopped and the tip discarded, before the analysis of the next sample began. The sample plate was kept at 15 °C throughout the acquisition. Samples were run in row order. The instrument was operated in full scan mode from *m/z* 150–1200 Da (for singly charged species).

### Liquid chromatography-mass spectrometry

LC-MS was run as we described in our recent studies [14, 16, 26, 27], with the additional use of CID for analyses described here. For all runs, chromatographic separation of lipid and triglycerides was achieved using a Waters Acquity UPLC CSH C18 (50 mm × 2·1 mm, 1·7 mm) LC-column with a Shimadzu UPLC system (Shimadzu UK Limited, Milton Keynes, UK). The column was maintained at 55 °C with a flow rate of 0·5 mL/min. A binary mobile phase system was used with mobile phase A; acetonitrile:water mix (6:4, respectively, with 10 mM ammonium formate), and mobile phase B; isopropanol:acetonitrile mix (9:1, respectively, with 10 mM ammonium formate). Gradient profile: 0 min, 40% mobile phase B; 0·4 min, 43% mobile phase B; 0·45 min, 50% mobile phase B; 2·4 min, 54% mobile phase B; 2·45 min, 70% mobile phase B; 7 min, 99% mobile phase B; 8 min, 99% mobile phase B; 8·3 min, 40% mobile phase B; 10 min, 40% mobile phase B. Mass spectrometry detection was performed on a Thermo Exactive orbitrap mass spectrometer (Thermo Scientific, Hemel Hempstead, UK). A heated electrospray source was used; the sheath gas was set to 40 (arbitrary units), the aux gas set to 15 (arbitrary units) and the capillary temperature set to 300 °C. The instrument was operated in full scan mode from *m/z* 100–1800 Da (for singly charged species). Lipid species were identified by detecting a signal peak for the corresponding accurate mass at the correct retention time. Signals were normalised to the total lipid/glyceride signal for that sample.

Signals data were collected across three runs. The first operating in positive ion and negative ion continuous switching mode. This was used for profiling the lipid fraction and quantifying all variables. The second run was a continuous negative ion mode switching between CID on and off. The third was a continuous positive ion mode switching between CID on and off. Runs 2 and 3 were used to identify the fatty acid composition of individual peaks in the chromatogram, in order to identify the configuration of the lipid isoform(s) present.

### Data handling and processing to produce a full lipidome

Both the direct infusion and liquid chromatography-mass spectrometry data are acquired in the form of .RAW files produced by the Thermo® Xcalibur software. Data from direction infusion mass spectrometry was processed through automated extraction of the relevant signals using an established in-house R code [25, 26, 28] and pre-determined search lists for the positive, negative and high energy negative (in-source fragmentation/collision-induced dissociation) ionisation modes used. This produced a list of signals with accompanying deviations. The first stage of data processing was to exclude signals that deviated too greatly from the expected high-resolution mass. The threshold used for deviations was informed by the deviations of the internal standards (ISs) used. Lipids used as internal standards perform well in at least one of the two modes; however, several are known to deviate more than an acceptable amount in one of the two modes. Thus, plotting the deviations of the IS lipids can be used to indicate the general deviation of that measurement period. Generally, a deviation cut-off of 9 ppm is sufficient for large (> 500) sample sets, where small ones (< 100) can be settled by a deviation of 5 ppm.

We then carried through variables whose signal intensity correlated with their concentration. This was determined through quality control (QC) samples of several types. We have developed a QC mixture that comprises a comprehensive range of commercially available mixtures of lipids from biological sources. We also used QCs of pooled human serum and mouse serum from the present samples, homogenised mouse livers, and a mixture of infant formulae and Jersey milk previously used [24, 29]. The variables that also had a signal-to-noise ratio (against IS-containing blank samples) of >3 were then identified. Variables with fewer than 25% values in any one sample group for each tissue were excluded.

### ^31^P NMR

Tissue homogenates were combined to give 5–10 mg of phospholipid per NMR sample. This approach was used as it is regarded by us as more generally representative of high-powered studies in which only a small quantity of material is available for each participant. The combined homogenates (~ 5 mL) were diluted with water (10 mL) and DMT (15 mL) in a Falcon tube (50 mL) before the mixture was agitated and centrifuged (3·2 k × *g*, 5 min). The bulk of the aqueous fraction and the mesophasic solid were discarded. The organic solution was concentrated to dryness (N_2 (g__)_) and the resulting film stored at −80 °C. Samples were dissolved in a modified [5, 30] form of the ‘CUBO’ solvent system [31–34] (the amount of dueteriated dimethylformamide *d*_7_-DMF was minimised, to around 20%). Stock solutions of the solvent consisted of dimethylformamide (3·5 mL), *d*_7_-DMF (1·5 mL), triethylamine (1·5 mL), and guanidinium chloride (500 mg). Wilmad® 507PP tubes were used. Sample concentration was 5–10 mg lipids per sample (600 μL).

Lipid samples were run on a Bruker Avance Neo 800-MHz spectrometer, equipped with a QCI cryoprobe probe. 1D Phosphorous experiments were acquired using inverse gated proton decoupling. Spectra were averaged over 1312 transients with 3882 complex points with a spectral width of 14·98 ppm. An overall recovery delay of 8·4 s was used. Data were processed using an exponential line broadening window function of 1·5 Hz prior to zero filling to 32,768 points and Fourier transform. Data were processed and deconvoluted using TopSpin 4.07. Subsequent integrations above a noise threshold of 0·01% of the total ^31^P signal integration were used to establish the molar quantity of a given phosphorus environment.

2D (^31^P-HSQC) spectra were acquired using an XY16-CMPG sequence for transfer and GARP-4 decoupling. The direct dimension consisted of 3072 points with 16 transients, and 2048 points in *t*_1_ collected using non-uniform sampling with a 25% sparsity, giving 256 points overall for the indirect dimension. Data were processed with compressed sensing, linear prediction in F1 and a shifted sine squared window functions.

### Statistical methods

Univariate and bivariate statistical calculations were made using Microsoft Excel 2016. Multivariate tests were carried out using MetaboAnalyst 4.0 [35]. We employed a Bonferroni correction for dependent variables (0·05/sqrt(*n*)) as the variables analysed were highly correlated, *e.g.p* = 0·0022 for 500 variables. Graphs were prepared in Origin 7.0 or Excel 2016 from mean and standard deviation.

## Results and discussion

### Design of the pipeline

The design of the pipeline incorporates several novel features for characterizing the lipid fraction of biological samples at the molecular level. This pipeline has been used on a set of mouse tissues (serum, adipose, vastus lateralis muscle, liver, kidney, brain, and heart) and two human sample types (serum, placenta) in order to indicate its capabilities and where special attention is required in applying the platform or individual steps to high-throughput studies.

#### Preparation of tissue samples

The texture and size of tissues, such as the vastus lateralis muscle and adipose, and organs such as the heart, liver, placenta and even brain can make handling awkward, especially if large numbers of samples are required for a high-throughput study. We have therefore developed a method for preparing pipettable liquid samples using a solution that is expected to suppress endogenous lipases and phosphatases [5, 36] and disrupt intermolecular reactions. We chose bladed homogenisation for ensuring physical disruption of the sample. It was found to be quicker and produced more consistent results than a pestle and mortar, and was suitable for all tissues. The contact areas of the blades were cleaned with organic solvents and then water after each sample homogenisation. Importantly, each homogenisation took place in the presence of a mixture of guanidinium chloride (6 M) and thiourea (1·5 M), referred to here as GCTU. The presence of these powerful chaeotropes during disruption ensures that cellular structures are dismantled at a molecular level and proteins are denatured readily, suppressing all enzymatic activity. This yielded typically translucent solutions that were readily pipettable.

The most fibrous tissue (vastus lateralis muscle) benefitted from freeze-thaw cycles in the appropriate buffer before physical disturbance. The addition of GCTU was not required for milk [24, 29], plasma or serum samples (data not shown). Samples containing particularly high concentrations of triglycerides produced biphasic systems in GCTU, with the upper phase containing most of the fat [24]. In order to ensure that the fattiest solutions were pipettable, we added 0·4 volumes of methanol and 0·2 volumes of *tert*-butylmethyl ether to favour formation of a monophasic solution shortly before sample transfer for extraction.

#### Extraction of the lipidome

The first widely accepted methods for extracting lipids from biological samples date from the 1950s and have been used countless times in the decades since [17]. Simplified forms of the procedures described by Folch et al. [37] and Bligh & Dyer [38] remain popular. However, since the chemical and physical behaviour of lipids has been characterised in detail a more nuanced understanding of them has emerged. This understanding suggests that exposing lipids to chloroform can result in chemical damage and that not all lipids of interest dissolve well in small quantities of chlorinated solvents (reviewed in [17]). These observations led us to test several improvements to the original procedures [5, 29, 30] that we have developed to produce a method that can be applied to high-throughput metabolic studies, described here for the first time.

In order to avoid the chemical damage associated with the use of chloroform [17] we have used dichloromethane in its place. We have also used triethylammonium chloride. This supplies a cation that is soluble in organic solvents and thus facilitates more polar lipids dissolving in the organic fraction. The extraction used also has a large enough volume of water to lower the concentration gradient of the guanidine and thiourea between the aqueous and organic phases. This therefore favours the transfer of lipids into the organic phase and other components into the aqueous phase.

These steps were incorporated into a procedure that can be used for extractions of the lipid fraction in a high-throughput manner. We used a 96-well plate format, with samples injected into the plate (well volume of 2·4 mL) before sequential addition of internal standards, water and a solution containing dichloromethane and triethylammonium chloride. We used a 96 channel pipette (liquid-handling robot, Integra Viaflow 96/384 channel pipette.), and designed the order of steps to minimise biohazard and plastic waste by using just one set of tips. The biphasic mixtures in each well were agitated in order to equilibrate the system, before centrifugation to sharpen the separation of the organic and aqueous phases. The bulk of the aqueous phase was removed to facilitate access to the (lower) organic solution. A portion of this was transferred to a mass spectrometry plate (typically 384 or 96 well) to profile the whole fraction.

We found subsequently that although this pipeline is satisfactory for most tissues, high-fat samples such as adipose tissue, and even jersey milk, typically give mass spectra dominated by triglycerides [24, 29]. This is useful for profiling the fatty components but it can make surveying the phospholipid fraction challenging as it is obscured by the abundance of triglycerides.

This led us to develop a method for removing the bulk of the triglyceride fraction from such samples in a non-selective manner. This was achieved by transferring a volume 5× greater than that used for the whole-sample profiling, drying and then washing with petroleum spirit or *n-*hexane. This dissolves the triglycerides but not the phospholipids, meaning they can be removed with relative ease. This reduces the abundance of triglycerides and increases the number of phospholipid variables observed [29].

#### Profiling of the lipid fraction using direct infusion mass spectrometry (DI-MS) with a CID mode

In order to produce a detailed molecular survey in a high-throughput manner, we have made use of Direct Infusion Mass Spectrometry (DI-MS) [25, 26, 28, 39]. In this process, samples in organic solution are injected into the Orbitrap without chromatography and ionised in positive and then negative modes. This has been used in a variety of studies of human metabolism [14, 26, 28, 40] as it enables sterols, lipids, and triglycerides to be profiled, despite very different ionisation efficiencies. Positive ionisation mode favours triglycerides (that adopt a positive charge through forming sodium or ammonium adducts) and phosphatidylcholines (PCs, that have a ‘permanent’ positive charge), where negative ionisation mode favours anionic phospholipids particularly, still with good signals from zwitterionic ones such as PC.

This two-part DI-MS method has been used successfully for several metabolomics studies [16, 25, 26]. In order to profile the fatty acids (FAs) in the phospholipid fraction, we have extended this to include a third mode that fragments species that ionise in negative mode (mainly phospholipids). This means the fatty acid profile can be determined readily and without the need for a second technique such as GC-MS [24, 29]. This is useful in metabolic studies as it can be used to identify changes in fatty acids associated with de novo lipogenesis (shorter, saturated species), and dietary intake, *e.g.* essential FAs. The three-part DI-MS method used to establish the lipidome in positive, negative and FA modes is very amenable to high-throughput studies, with it being possible to run a 384 well plate in only 32 h [24, 29].

#### Profiling a subset of samples using liquid chromatography-mass spectrometry with CID

Liquid chromatography-mass spectrometry (LC-MS) was used to verify the signals identified in DI-MS [14, 26, 27, 39] and exploit in-source fragmentation (CID) in order to identify the fatty acids associated with individual lipid variables. Verification of the existence of signals observed in DI-MS using LC-MS is scientifically valuable as it provides further evidence of the molecular structure of the species identified. It represents a targeted approach after an untargeted one, in order to acquire detailed information of a smaller number of species of greater importance to the hypothesis being tested.

Signals data were collected across three runs. The first operating in positive ion and negative ion continuous switching mode. This was used for profiling the lipid fraction and quantifying all variables. The second run was a continuous negative ion mode switching between CID on and off. The third was a continuous positive ion mode switching between CID on and off. Runs two and three were used to identify the fatty acid composition of individual peaks in the chromatogram, associated with a known variable, in order to identify the configuration of the lipid isoform(s) present. A combination of the positive and negative-CID runs was used to determine the fatty acid profile of isoforms identified in DI-MS and confirmed by LC-MS. An example of this is shown in Fig. S1 (see ESM).

PC(36:6) was identified as being present only in the kidneys of obese mice. This raises metabolic questions about why a polyunsaturated lipid should be there, but also what the configuration is. The precise configuration may shed light on how obese systems (mis)manage the distribution of PUFA-containing lipids. We used a combination of LC-MS with both positive ionisation and negative ionisation mode with CID switching to identify fragments at the appropriate retention time, for this lipid. This showed clearly that PC(36:6) was PC(14:0/22:6) (ESM Fig. S1). This suggests that in obese systems, at least one essential fatty acid containing lipid is transported to the kidney in a way that does not occur in lean mice.

We also used this approach to quantify how a mixture of configurations differs according to phenotype. PC(38:5) is evident in the hearts of both lean and obese mice. However, a number of configurations are possible. We found that this lipid consists of trace amounts of PC(16:0/22:5), with most of the signal being ascribed to either PC(20:3/18:2) or PC(20:2/18:3). However, the proportion appears to change, with PC(20:3/18:2) being around 10% more abundant in the hearts of obese mice than lean ones. This approach therefore offers a way of characterising the lipid profile of different phenotypes and tissues in detail.

#### Signal identification using dual spectroscopy

Mass spectrometry has proved a very useful tool in lipidomics and will remain the main technology used in this discipline for high-throughput studies. It is possible to acquire large amounts of information and quantify the abundance of hundreds of lipids from relatively small samples using this technique. The availability of three ionisation modes as shown here has extended the range of species that can be observed in one DI-MS method. However, inherent in this technique is that positive and negative ionisation modes offer different ionisation efficiencies. Triglycerides and phosphatidylcholines ionise efficiently in positive ionisation mode and anionic phospholipids, especially *lyso*-lipids ionise particularly well in negative ionisation mode.

This can however make accurate quantification of lipid variables difficult and restricts automation of the process. Furthermore, there is no standard approach to signal identification. Some approaches are relaxed, meaning the bulk of the ‘true’ signals are identified, but also noise can be incorrectly identified as a signal. There appear to be several examples of this in the recent literature, with quite unrealistic assignments being made in some cases. Some approaches are more conservative, in which mis-identification is much less common but where real signals may be missed. Neither of these approaches is objectively better as they both risk arriving at an incorrect answer.

In this study, we have addressed questions about reliable signal identification and ionisation efficiency of lipid classes by using ^31^P NMR. This second, orthogonal, technique shows the relative abundance of the chemical environments of the phosphorus atom in the phospholipid head group and thus gives an insight into the relative ionisation efficiency of lipid head groups in mass spectrometric measurements. This technique is therefore a useful ‘reality check’. The technique comes with its own limits, one of which is that considerably more material is required for a ^31^P NMR sample than an MS one. This means that in most high-powered studies, several samples from each phenotype or group should be pooled to produce a large enough sample for practical ^31^P acquisition. The approach of using two or more orthogonal techniques for structural determination has been common practice in synthetic chemistry for some years, but has entered lipidomics more recently [5, 30]. Dual spectroscopy therefore offers a circumspect but well-supported approach to lipidomics.

An independent measure of the ratio of the relative abundance of head groups can be useful for both distinguishing gross changes between phenotypes as well as for producing ‘rules of thumb’ in assigning IDs to signals in mass spectrometry. Even marginal differences in phospholipid abundance between groups can be observed using ^31^P NMR. This is indicated by a comparison of lean and obese kidney lipid extracts (ESM Fig. S2). One clear rule of thumb comes from serum samples. ^31^P NMR reveals that in serum, phosphatidylethanolamine (PE) has an abundance around two orders of magnitude lower than that of phosphatidylcholine (PC, ESM Fig. S3). This suggests that an abundant variable with an *m/z* consistent with a PE should either be given an alternative assignment (is an isobar) or ionises better than the expected variable. For example, when a signal that represents the *m/z* for both an isoform of phosphatidylethanolamine, PE(38:2) and one of phosphatidylcholine PC(35:2) is recorded (*m/z* 772·5851, proton adduct) in a serum sample, the balance of probabilities rests with it being an isoform of PC rather than a PE, even for relatively low-abundance configurations that comprise a fatty acid residue with an odd number of carbons. This can be supported by analysis using mass spectrometry in negative ionisation mode. PEs ionise easily as they can be deprotonated to form a negatively charged species. However, PCs have a ‘permanent’ positive charge by virtue of their quaternary amine, a feature that must be masked before it can adopt an overall negative charge. This confers a difference in mass on ionised PCs and PEs in negative mode, meaning they can be distinguished.

^31^P NMR was used to gain further insight into the profile of lipid classes in the samples of interest and even some of the phenotypes (see Figs. S2, S3, S9, and supplementary spectra in ESM), and was used to inform the mass spectra used in the present study. This included respect for the abundance of PE with respect to isobaric PC but also the abundance of lipids such as phosphatidic acid, and phosphatidylserine and phosphatidylglycerol.

The ability to assign signals to resonances is essential for the successful use of ^31^P NMR in lipidomics. The existence of more than one resonance for PE in the present solvent system has been reported at least three times [5, 30, 34], described as being due to interaction of this lipid with ion is in the solvent system (triethylammonium, guanidinium, and metal ions being the most likely). We therefore used two-dimensional NMR (^31^P-HSQC) to look further into the structures of the resonances observed in an attempt to assign them more accurately. This analysis (ESM Fig. S4) indicated that PE does indeed have several phosphorus resonances (0·55, 0·30, 0·15 ppm) with plasmalogen PC located at around 0·05 ppm, contrary to some previous reports. Furthermore, investigation using ^31^P-HSQC suggested to us that there may also be more than one resonance for PC, though with more similar shifts than are observed for PE such that they are not typically separated, *e.g.* mouse kidney samples (0·0 ppm, see Fig. S2 and supplementary spectra in ESM). This approach may be useful to interpret otherwise unclear spectra and thus contribute towards an accurate phospholipid profile.

#### Lipidome analysis

Semi-quantitative and quantitative lipidomics data on a variety of tissues from a group of individuals associated with a particular phenotype (such as obesity) offers a unique opportunity to characterise that phenotype. In particular, the locus of changes in lipid metabolism can be rapidly assessed in a way that is not possible from serum samples alone. Here, we discuss three simple analyses for determining differences between phenotypes: (a) abundance analysis, (b) fatty acid biosynthesis, (c) candidate biomarker discovery.

Simple plots of abundance can be used as a tool for determining which tissues are of principal interest for a given phenotype. For example, in the present mouse obesity phenotype, liver is changed considerably (ESM Fig. S5A) but brain appears to be little changed (ESM Fig. S5B). Specifically, profiles of the liver of lean and obese post-weaning dams showed that the abundance of triglycerides was much higher in tissues from obese individuals. We therefore suggest that this calculation is useful for identifying the tissues of greatest interest for characterizing the lipid metabolism in a particular phenotype.

**Fig. 1:**
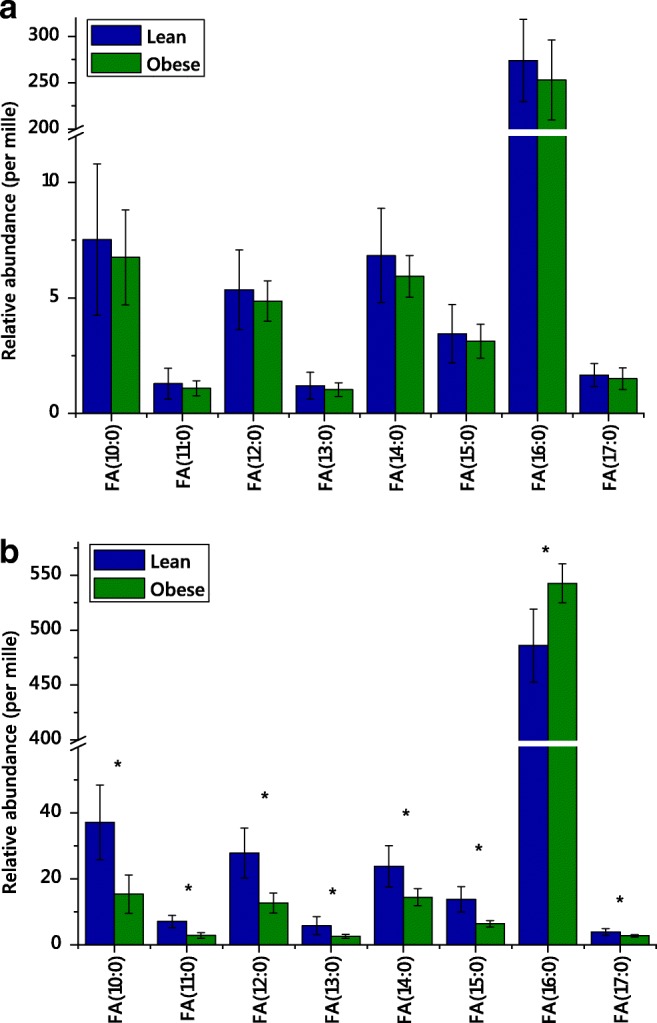
The fatty acid residues from phospholipids from the brains (top panel, **a**) hearts (bottom panel, **b**) of lean and obese mice. All variables differed significantly in hearts (marked *, false-discovery rate-adjusted *p* value based on Bonferroni calculation with dependent variables), but none did in brains. Fatty acids detected and quantified (relative quantification) measured using mass spectrometry in negative ionisation mode with collision-induced dissociation. Error bars show standard deviation

An abundance analysis using values across samples, for example of fatty acids, can also be revealing. The fatty acid profile of phospholipids was acquired using DI-MS (*vide supra*) though negative ionisation with CID. We found that several fatty acids differed in abundance in the hearts of lean and obese dams (Fig. 1). These results are interesting for metabolic study as they suggest that FA(16:0), an established marker of de novo lipogenesis (DNL), is more abundant in obese mice. This suggests that DNL is different between these two systems and that the lipid composition of the heart reflects this. This analysis also uncovers the pattern that odd-chain fatty acid residues are more abundant in the hearts of lean mice. These results are interpretable in terms of the aetiology of metabolic and heart disease [24, 41–44] in a manner that is orthogonal to the abundance analysis of whole lipids.

Abundance analysis can be used to indicate general changes in lipid metabolism. The same lipid data from several tissues from the same individual with a particular phenotype that are analysed simultaneously can also be used to identify changes in the activity of individual enzymes associated with lipid metabolism. For example, comparing the ratio of particular lipids to one another can be used to infer the activity of particular enzymes involved in fatty acid biosynthesis and modification. A variety of lipid abundance ratios have been used for this, either using the ratio of particular fatty acids or lipids that comprise them: fatty acid elongase (FA(18:0)/FA(16:0); PC(36:4)/PC(34:2)) [45–47], Stearoyl co-acetyl desaturase (CE(16:1)/CE(16:0); CE(18:3)/CE(18:2); PC(32:1)/PC/32:0)) [48, 49], fatty acid desaturase 1 (FADS1, PC(38:4)/PC(38:3); TG(54:4)/TG(54:3)) [45, 46], and fatty acid desaturase 2 (FADS2, PC(36:3)/PC(36:2); TG(50:3)/TG(50:2)) [45, 46].

We calculated the ratio of TG(50:2) to TG(50:3) for both lean and obese mice as a marker of FADS2 activity (ESM Fig. S6). This showed that the ratio of these two lipids was similar in the adipose of lean and obese mice. However, lipid profiles from brain and liver show that the ratio of TG(50:2) to TG(50:3) was higher in lean mice than in obese ones, with the opposite true in kidney, serum, heart, and vastus muscle. This suggests that FADS2 activity is modulated in obese individuals.

Discovery of candidate biomarkers (CBMs) is an established technique for identifying the variables that both distinguish two groups and drive the statistical difference between them [13, 16, 24, 26]. This involves running a sparse partial least squares discriminant analysis [35] followed by a student’s *t* test with a significance threshold adjusted for multiple variables and/or comparisons as appropriate.

Most studies in which CBM discovery has been employed have used plasma or serum samples. We tested our lipidomics pipeline on a modest set of human sera from pregnant (*n* = 12, 28 weeks gestation) and non-pregnant (*n* = 10) women at both the fasting and 2 h time points of the oral glucose tolerance test (OGTT). The 2 h time point is the time after ingesting glucose (75 g). The frozen serum samples were thawed and the lipid fraction extracted from samples in a high-throughput manner, followed by DI-MS. Paired analyses of the fasting and 2 h samples of both the pregnant and non-pregnant groups suggested that just one variable, *m/z* 824·5447 (negative ionisation mode), differed significantly between the groups. This *m/z* is ascribed either to PC(36:5) with a formate adduct, PC(35:5) with an acetate adduct or possibly deprotonated PS(39:4) in negative ionisation mode. This subtle difference between 0 h and 2 h samples is reflected in the comparison between pregnant and non-pregnant samples made by a Principal Component Analysis (ESM Fig. S7). The latter analysis indicated that pregnant and non-pregnant systems differ considerably in their lipid metabolism, consistent with previous work [50, 51], that the variables that distinguished fasting and postprandial samples from pregnant participants are different to those that distinguish fasting and postprandial samples from non-pregnant women (PC2).

However, (unpaired) comparisons of pregnant and non-pregnant groups at fasting and 2 h showed that a considerable number of variables differed in abundance between the two groups (ESM Fig. S8). This suggests that even with relatively low statistical power, a number of trends in lipid metabolism that distinguish pregnant and non-pregnant women are visible (ESM Fig. S8). This includes a general shortening of the chain lengths of fatty acids in phosphatidylglycerol (PG), a shortening of fatty acid chains and reduction in the number of unsaturated bonds in both PEs and PC plasmalogens. The abundance of isoforms of phosphatidylinositol (PI) associated with signalling inositides is reduced in the circulation of pregnant women. The ^31^P NMR results (ESM Fig. S3) show that there is an increase in the relative abundance of (minor lipids) PI and plasmalogen PC, and a more marked increase in sphingomyelin (SM), but that PC is still dominant and has the same ratio with *lyso*-PC in both phenotypes. There is little evidence of a major change in the overall relative abundance of PE. This suggests that although the PEs appear abundant in negative mode, and the fatty acid profile of the PE fraction changes significantly, the overall abundance of PE, PC, and *lyso*-PC does not change markedly.

**Fig. 2:**
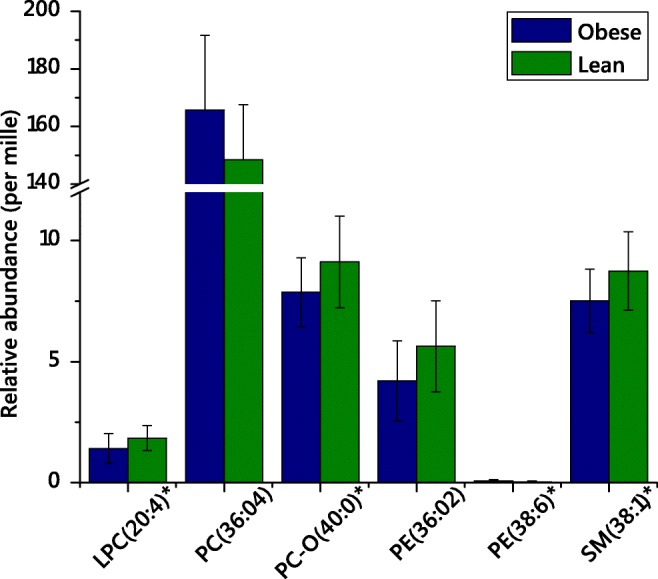
Candidate biomarkers that represent differences in lipid metabolism in lean and obese placentae, from term singleton pregnancies collected in the Pregnancy Outcome Prediction Study. Variables shown represented those that distinguished the two phenotypes (lean/obese) and were significant (pass at false-discovery rate-adjusted *p* value [Bonferroni FDR correction for many dependent variables])

The interest in the role of lipid metabolism in human development has led to an interest in extracting and profiling lipids from other human tissues, including more fibrous ones such as placenta. We sought to characterise the shift in metabolism associated with obesity in mothers by searching for biomarkers that differentiate human placenta samples. Candidate biomarker discovery found that six lipid species both differed significantly (*p* value corrected for multiple variables, based on 455 variables) and were identified as distinguishing the two phenotypes using an sPLS-DA, all of which were phospholipids (Fig. 2).

This approach indicates that two polyunsaturated lipids comprising two fatty acid residues (PC(36:4), PE(38:6)) are less abundant in lean placentae. This is remarkable as PC(36:4) represents ~ 15% of the total lipidome as measured in positive ionisation mode. Saturated or *mono*-unsaturated-containing species (PC-O(40:0), PE(36:2), SM(38:1)) are more abundant. *lyso-*PC(20:4), a possible degradation product of PC(36:4) and marker of a variety of metabolic changes, is also more abundant in obese placentae. These observations indicate that the metabolism of phospholipids, at least one of which can be regarded as very abundant in this lipidome, is altered in obesity.

These data indicate that in general, lipidomics of membranes in obese placentae differ across at least two lipid biosynthesis pathways. This modulation in composition raises questions about the physical behaviour of such membranes. Studies of the mesophase behaviour of lipid systems [52] and of the role of PE in biological systems [53] suggest that changes to PC and PE composition can have profound effects on the behaviour of such systems. The higher abundance of the fluidising PC(36:4) in obese placentae suggests that membranes in those placentae may be more fluid than those in lean ones. However, the ratio of total PE to PC indicates that there is in general more PE in lean placentae (measured in positive ionisation mode, *p* = 0·0003). This is reflected in pooled human placenta samples subjected to ^31^P NMR, where the abundance of PE appears to be slightly higher in lean placentae (ESM Fig. S9). This suggests that the biophysical behaviour as well as the lipid metabolism differs in lean and obese human placentae. Furthermore, ^31^P NMR also indicates that globally, there may also be slightly more PI and SM in lean placentae, also expected to have an impact on mesophase behaviour [54, 54]. This type of analysis, on a pilot-sized group (*n =* 79), can be used to inform how lipid metabolism in placentae is investigated in a larger sample set.

## Conclusions

The platform described here provides methods that can be used individually in existing procedures or in series to facilitate characterisation of the lipid fraction of biological samples. The analysis of a set of mammalian tissues from lean and obese mice demonstrates that the access to the lipid fraction of a variety of tissues enables researchers to answer questions and test hypotheses that hitherto had too high an experimental barrier to overcome. This is particularly useful for metabolic studies as it allows the effect of phenotypes such as obesity to be understood in different organ systems, and thus compared. The depth and breadth of this approach to lipidomics paves the way for the development of bioinformatics tools focused on lipid metabolism.

## Electronic supplementary material

ESM 1 (PDF 975 kb)

ESM 2 (PPTX 4.16 mb)

ESM 3 (XLSX 616 kb)

ESM 4 (XLSX 810 kb)

ESM 5 (XLSX 350 kb)

ESM 6 (XLSX 199 kb)

ESM 7 (XLSX 160 kb)

ESM 8 (XLSX 86 kb)

ESM 9 (XLSX 227 kb)

ESM 10 (XLSX 131 kb)

ESM 11 (XLSX 102 kb)

ESM 12 (DOCX 1.48 mb)
