# Supplementary material for: A high-throughput platform for detailed lipidomic analysis of a range of mouse and human tissues
Source: Anal Bioanal Chem. 2020 Mar 7;412(12):2851–62. doi: 10.1007/s00216-020-02511-0 (PMC7196091; doi:10.1007/s00216-020-02511-0)
Supplement: Supplementary file 1 — (PDF 975 kb) [file 216_2020_2511_MOESM1_ESM.pdf]

Analytical and Bioanalytical Chemistry

Electronic Supplementary Material

A high throughput platform for detailed lipidomic analysis of a range of mouse and human tissues

Samuel Furse, Denise Fernandez-Twinn, Benjamin Jenkins, Claire L. Meek, Huw E. L. Williams, Gordon C. S. Smith, D. Stephen Charnock-Jones, Susan E. Ozanne, Albert Koulman

Additional files available under 10.1007/s00216-020-02511-0.

Content

Supplementary Tables

Table S1 – Participant information for human serum from pregnant and non-pregnant women

Table S2 – Participant information for Prediction Outcome Prediction (POP) Study

Table S3 – Preparation of tissue samples for extraction of the lipid fraction

Table S4 – Internal standards used

Supplementary Figures

Figure S1 – LC-MS used to determine the FA profile of phospholipid variables

Figure S2 – 31P NMR used to investigate the shift in phospholipid profile between kidneys of lean and obese mice

Figure S3 – 31P NMR used to investigate the shift in phospholipid profile between pregnant and non-pregnant samples

Figure S4 – 2D-31P NMR used to investigate the configuration of lipids associated with 1D 31P resonances

Figure S5 – Phospholipid variables unique to the livers and brains of either obese or lean individuals

Figure S6 – Activity of FADS2 through ratio of abundance of TG(50:3) with TG(50:2) from lean and obese mouse tissues

Figure S7 – Unsupervised multivariate analysis of the lipid profile of sera from pregnant and non-pregnant women

Figure S8 – Abundance of phospholipids that change significantly between pregnant and non-pregnant women at fasting at 2 h post prandial

Figure S9 – 31P NMR used to investigate the shift in phospholipid profile between lean and obese placenta samples

Separate Files

Representative 31P NMR spectra with deconvolutions (PPTX)

Signals files of mass spectrometry data for all tissue samples used in the present study (9 * XLSX)

Supplementary Tables

Table S1 Participant information for human serum samples from pregnant and non-pregnant women

	Non-pregnant	Pregnant*	Significance
n	12	15	
Age (y)	28.50	29.25	0.84
BMI	23.70	24.80	0.53
BGC fasting (mM)	5.12	4.13	4.78E-05
BGC +2 h (mM)	6.36	5.43	0.10
HbA_{1C}	34.20	30.73	0.04

*OGTT at 28 weeks' gestation

BGC, blood glucose concentration; BMI, body mass index (based on weight (kg)/height (m) squared). +120 min refers to samples that were taken a number of minutes after ingestion of glucose (75 g) at fasting.

Table S2 Participant information for human placenta samples from Pregnancy Outcome Prediction Study

	Lean	Obese	Significance
n	39	40	-
Age (y)	30.77	29.73	0.026
BMI	33.37	21.06	5.6×10^{-25}
Male offspring (%)	58	28	-

Villious sections (30-40 mg) of placentae from vaginal singleton births.

Table S3 Preparation of tissue samples for extraction of the lipid fraction

Tissue	Mass (mg)	GCTU (mL)	Freeze-thaw	Homogenisation	Storage	Further dilution	Vol. used in lipid extraction (µL)
Human serum	n/a	0	0	-	-80 °C	n/a	20
Mouse serum	n/a	0	0	-	-80 °C	n/a	20
Mouse adipose	~250	2	n/a	TissueRuptor II	-80 °C	Methanol (400 µL) and TBME (100 µL)	8
Mouse brain	~450	2	n/a	TissueRuptor II	-80 °C	n/a	30
Mouse kidney	~200	1	n/a	TissueRuptor II	-80 °C	n/a	50
Mouse liver	~400	2	n/a	TissueRuptor II	-80 °C	n/a	10
Mouse heart	~100	1	1×	Pestle & mortar*	-80 °C	n/a	50
Mouse vastus muscle	~150	0.6	3×, 1× after homog.**	TissueRuptor II†	-80 °C	n/a	50
Human placenta (portion)	30-40	0.7	n/a	TissueRuptor II	-80 °C	n/a	50

*Before being dispersed in GCTU, samples ground to a powder at -78 °C. **Samples were checked for the presence of further solid material after freeze-thawing, those with solid material were homogenized again. † Samples were centrifuged briefly after homogenization, before being frozen (<20 s, up to 5k × *g*).

Table S4 List of internal standards used for lipid profiling in the present study

**Lipid Class**	**Isoform**
Cholesteryl ester	CE(18:0-d_6)
Ceramide	C16-d_{31} Ceramide
Fatty acid	C15:0-d_{29} FA
Fatty acid	C17:0-d_{33} FA
Fatty acid	C20:0-d_{39} FA
lyso-Phosphatidylcholine	lysoPC(C14:0)-d_{42}
Phosphatidic acid	PA(C16:0-d_{31}/C18:1) Na^+ salt
Phosphatidylcholine	PC(C16:0-d_{31}/C18:1)
Phosphatidylethanolamine	PE(C16:0-d_{31}/C18:1)
Phosphatidylglycerol	PG(C16:0-d_{31}/C18:1) Na^+ salt
Phosphatidylinositol	PI(C16:0-d_{31}/C18:1) NH_4^+ salt
Phosphatidylserine	PS(C16:0-d_{62}) Na^+ salt
Sphingomyelin	SM(C16:0-d_{31})
Triglyceride	TG(45:0-d_{29})
Triglyceride	TG(48:0-d_{31})
Triglyceride	TG(54:0-d_{35})

Supplementary Figures

LC-MS used to determine the FA profile of phospholipid variables

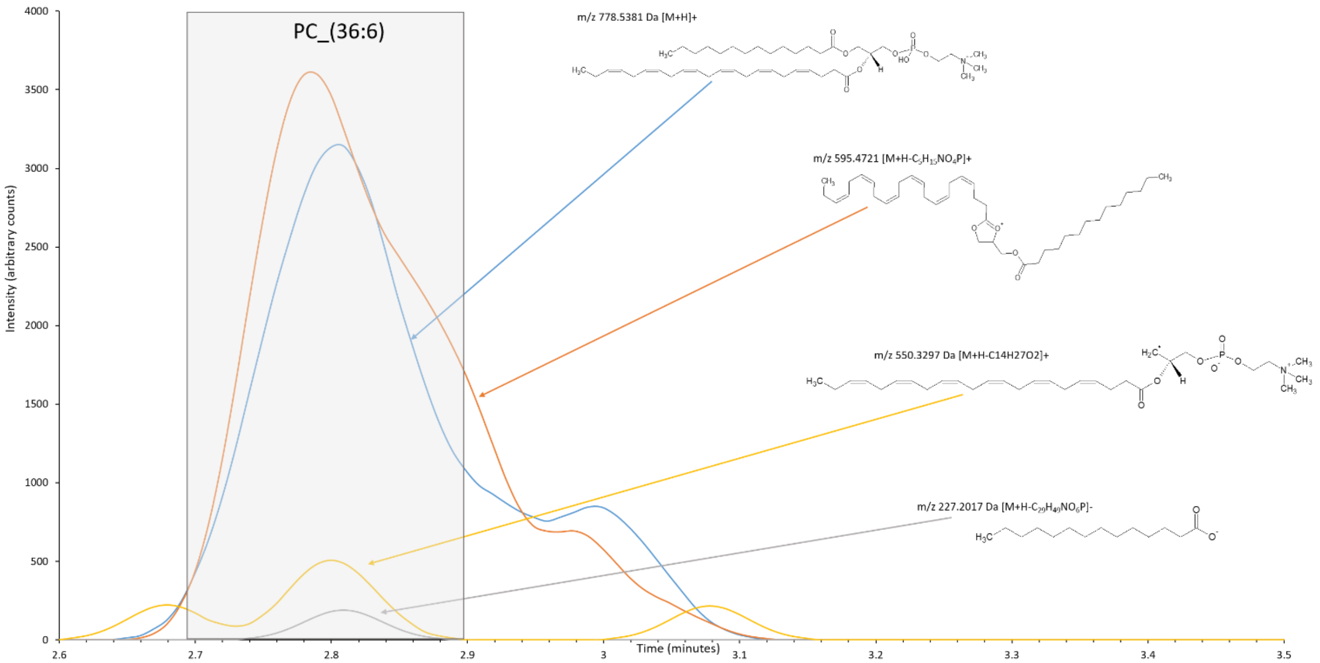

Fig. S1 Analysis of PC(36:6) using LC-MS in both positive and negative ionization modes with collision induced dissociation (CID): positive/positive CID switching and negative/negative CID switching. Fragments observed across both modes are shown

^{31}P NMR used to investigate the shift in phospholipid profile between kidneys of lean and obese mice

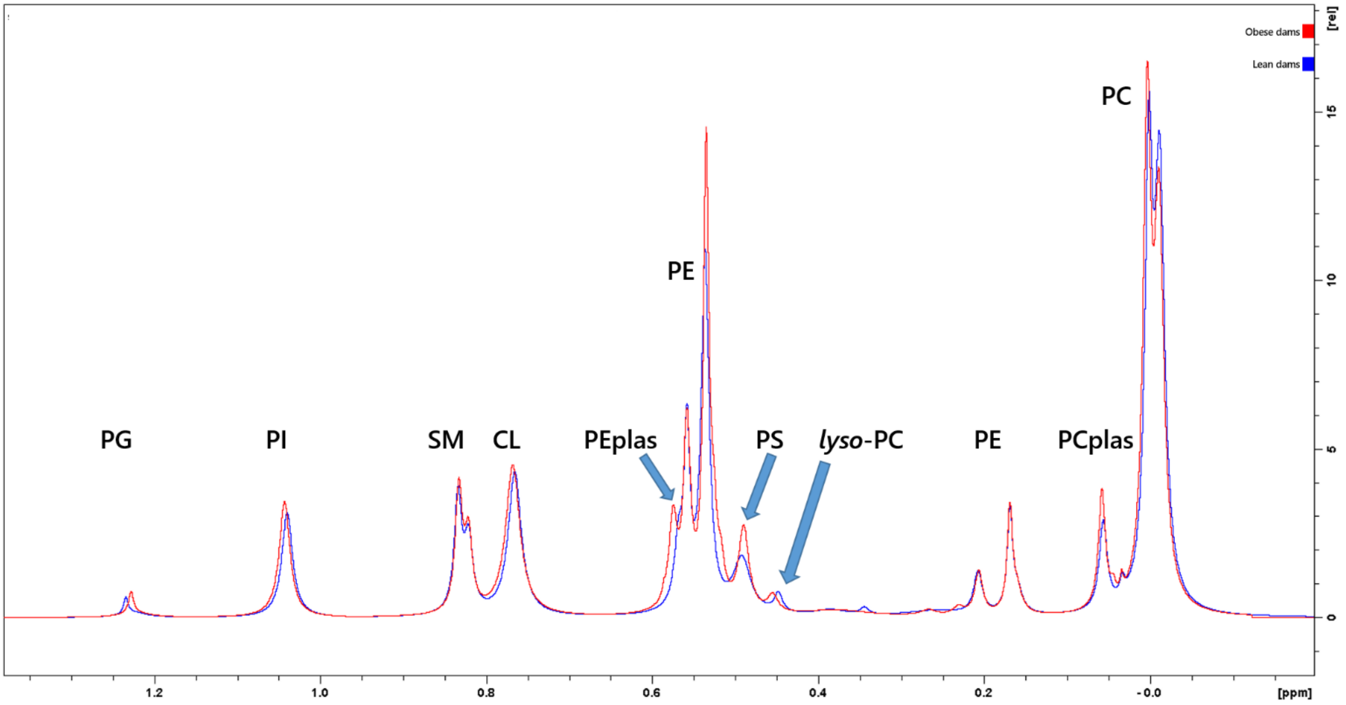

Fig. S2 Stacked, deconvoluted, 1D ^{31}P NMR spectra from pooled mouse kidney samples collected from lean and obese post-weaning dams. Phosphatidylethanolamine signals appear at 0·56, 0·53, 0·21, 0·17 ppm (See Fig. S4)

^{31}P NMR used to investigate the shift in phospholipid profile between pregnant and non-pregnant samples

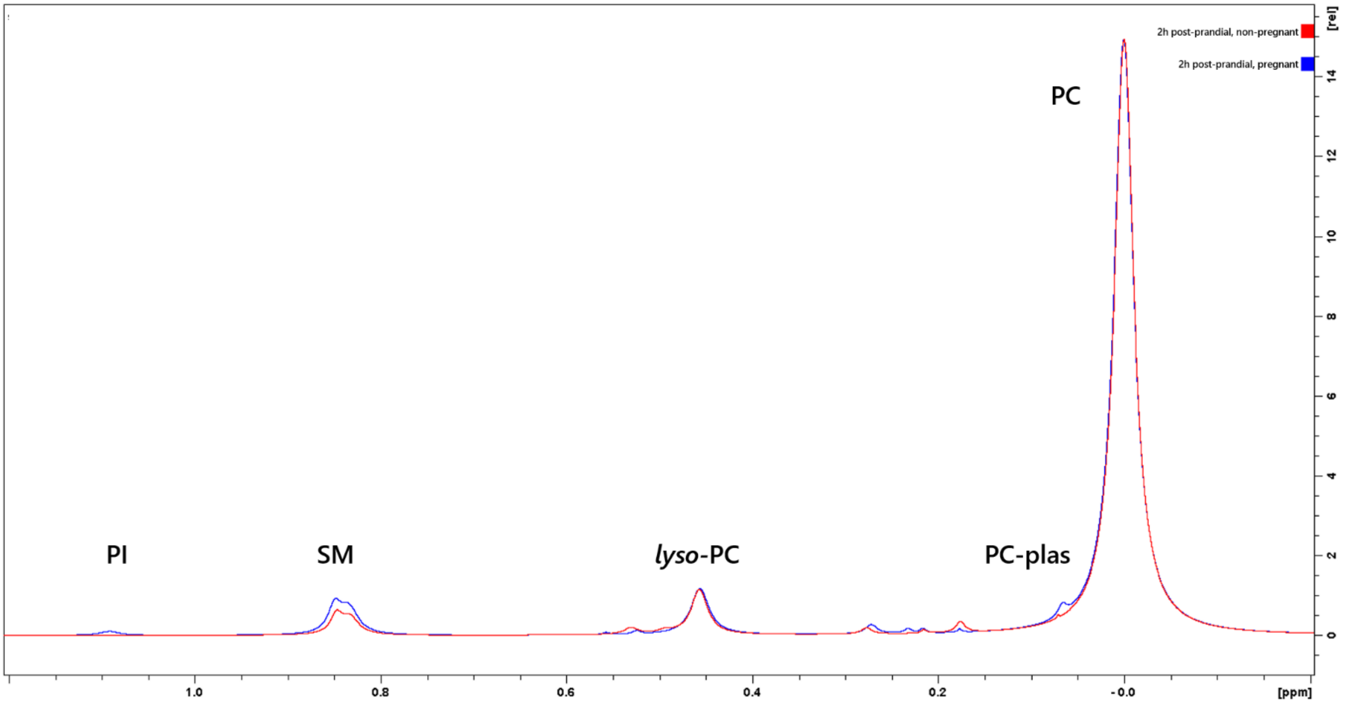

Fig. S3 Stacked, deconvoluted, 1D ^{31}P NMR spectra from pooled human serum samples collected from pregnant and non-pregnant women during an oral glucose tolerance test. Phosphatidylethanolamine signals appear at 0·55, 0·28, 0·18 ppm (See Fig. S4)

2D-^{31}P NMR used to investigate the configuration of lipids associated with 1D ^{31}P resonances

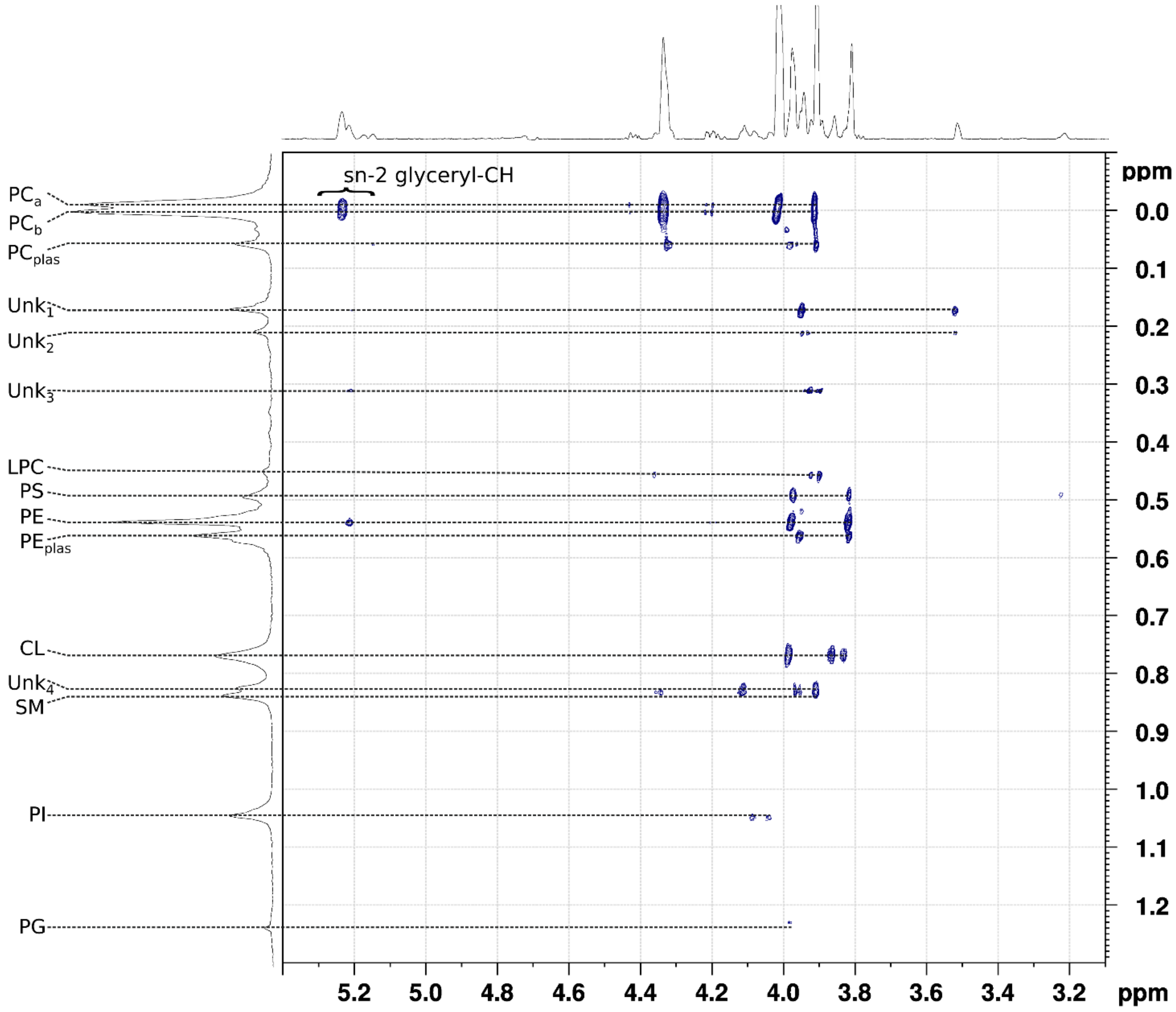

Fig. S4 ^{31}P-HSQC spectrum of kidney from obese post-weaning mouse dams. This suggests that phosphatidylethanolamine signals appear at 0·56, 0·53, 0·21, 0·17 ppm (Unk$_1$, Unk$_2$), that PCplas has a shift of 0·05 ppm and that the resonances for SM and PC can be split as well as those for PG and PE. CL, cardiolipin; LPC, *lyso*-phosphatidylcholine; PC, phosphatidylcholine; PCplas, plasamalogen-phosphatidylcholine; PE, phosphatidylethanolamine; PEplas, plasmalogen-phosphatidylethanolamine; PG, Phosphatidylglycerol; PI, phosphatidylinositol; PS, phosphatidylserine; SM, sphingomyelin

Phospholipid class abundance in the livers and brains of obese and lean mice

A

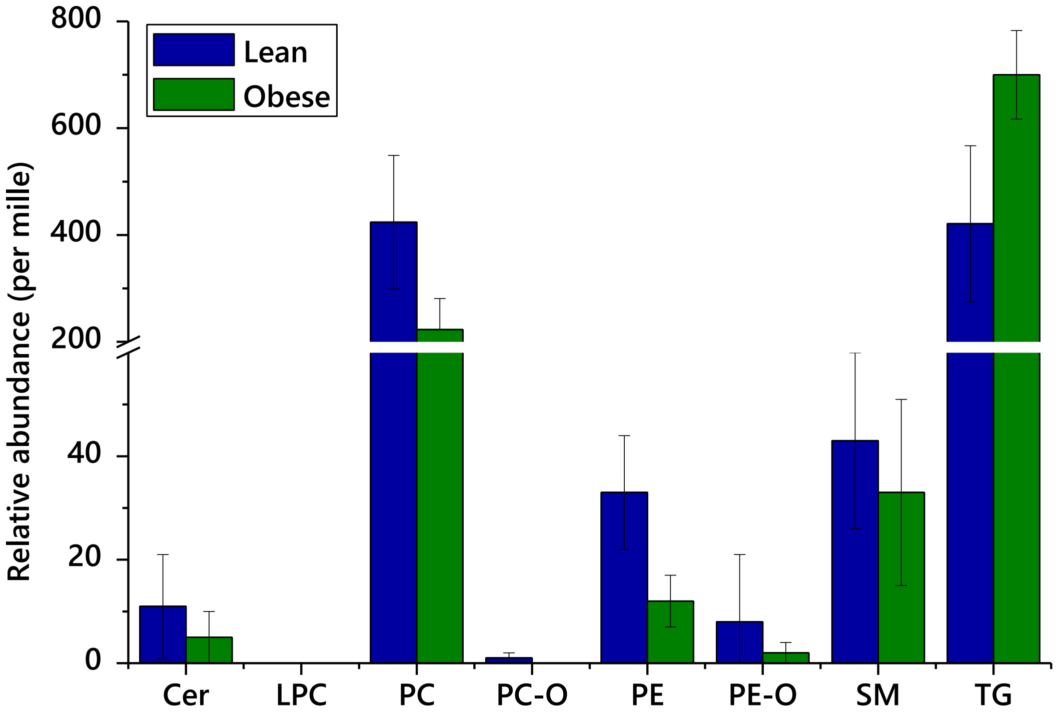

B

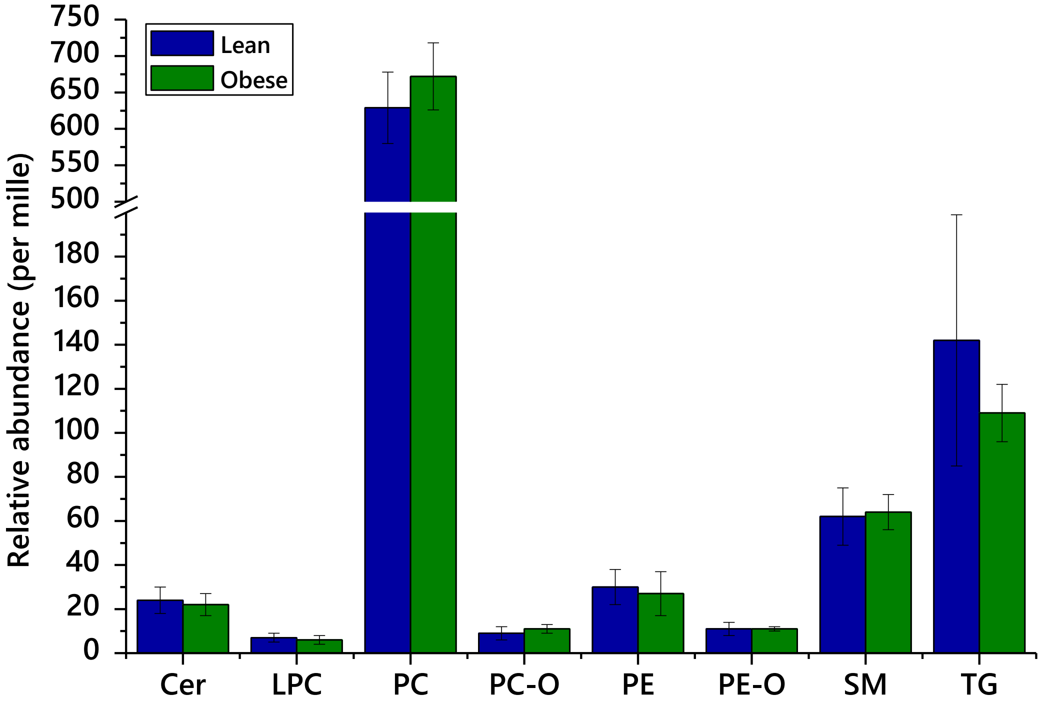

Fig. S5 The average relative abundance of phospholipid classes in the livers (top) and brains (bottom) of lean and obese groups of mice. Measured by positive ionisation mode mass spectrometry. Error bars show standard deviation

Activity of FADS2 through ratio of abundance of TG(50:3) with TG(50:2) from lean and obese mouse tissues

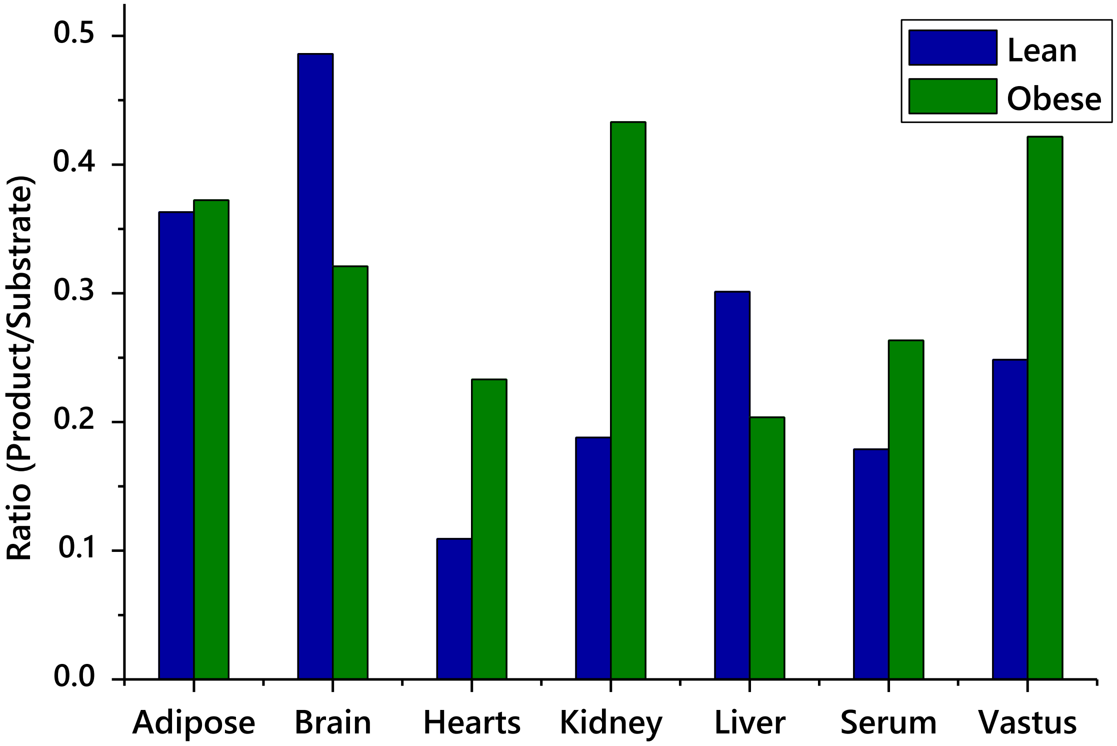

Fig. S6 The ratio of TG(50:03)/ TG(50:02) in tissues from lean and obese mice. Values calculated from the mean relative abundance of the TG(50:03) divided by the mean relative abundance of TG(50:02) (see signals sheets for calculations). This ratio implies the activity of fatty acid desaturase 2 (FADS2).

Unsupervised multivariate analysis of the lipid profile of sera from pregnant and non-pregnant women

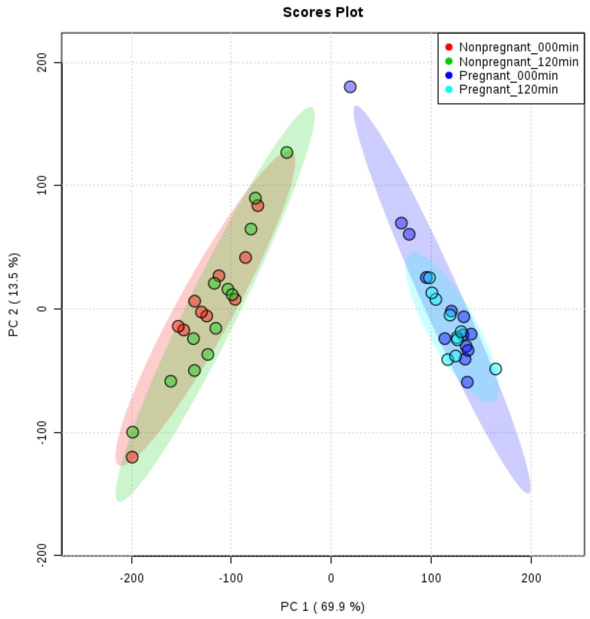

Fig. S7 Principal component analysis of the lipid profiles of serum samples from pregnant and non-pregnant women both at fasting (000 min) and 120 min after ingestion of glucose (75 g)

Abundance of phospholipids that change significantly between pregnant and non-pregnant women at fasting at 2 h post prandial

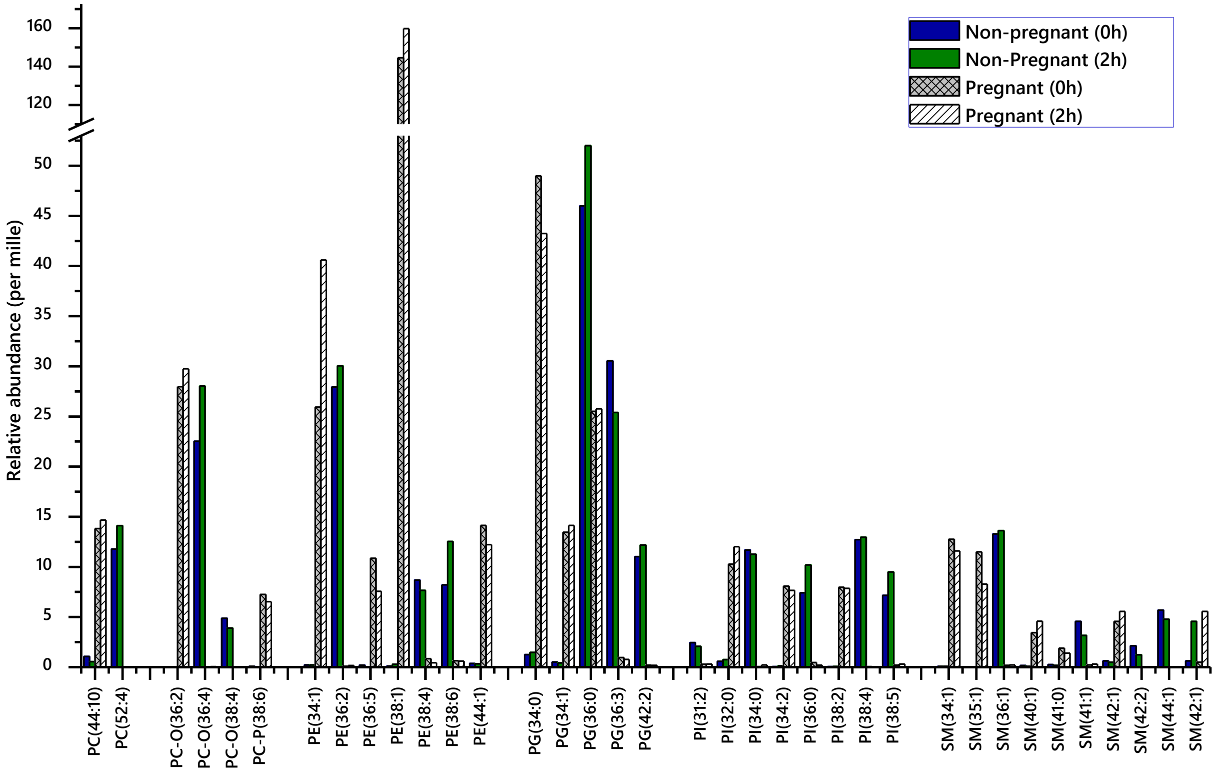

Fig. S8 Abundance of lipids in serum samples from pregnant and non-pregnant women both at fasting (0 h) and 2 h after ingestion of glucose (75 g), measured in negative ionization mode

^{31}P NMR used to investigate the shift in phospholipid profile between lean and obese placenta samples

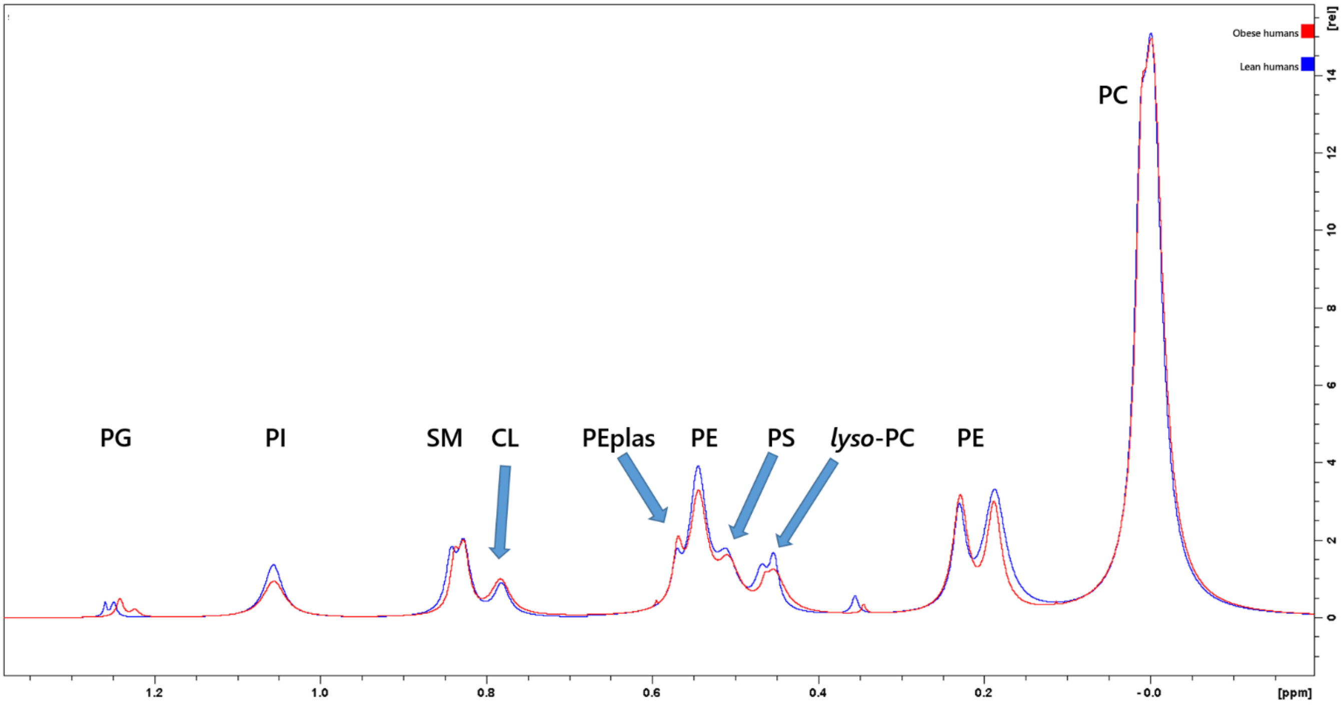

Fig. S9 Stacked, deconvoluted, 1D ^{31}P NMR spectra from pooled human placentae samples collected from lean and obese vaginal singleton male births. Phosphatidylethanolamine signals appear at 0·54, 0·24, 0·18 ppm, See Fig. S4
