# Supplementary material for: A high-throughput platform for detailed lipidomic analysis of a range of mouse and human tissues
Source: Anal Bioanal Chem. 2020 Mar 7;412(12):2851–62. doi: 10.1007/s00216-020-02511-0 (PMC7196091; doi:10.1007/s00216-020-02511-0)
Supplement: Supplementary file 2 — (PPTX 4.16 mb) [file 216_2020_2511_MOESM2_ESM.pptx]

## Slide 1
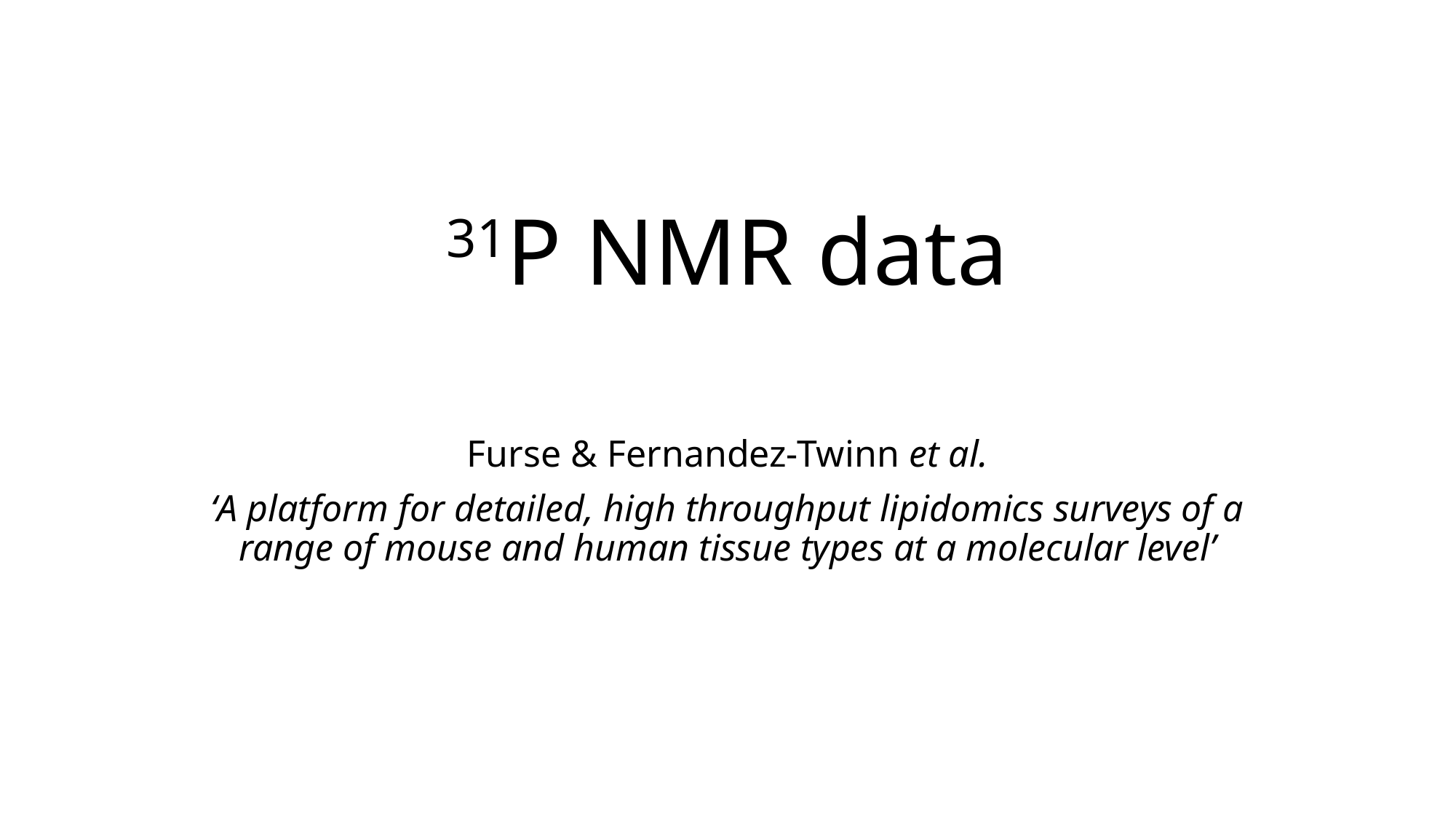

# 31P NMR data
Furse & Fernandez-Twinn et al.
‘A platform for detailed, high throughput lipidomics surveys of a range of mouse and human tissue types at a molecular level’

## Slide 2
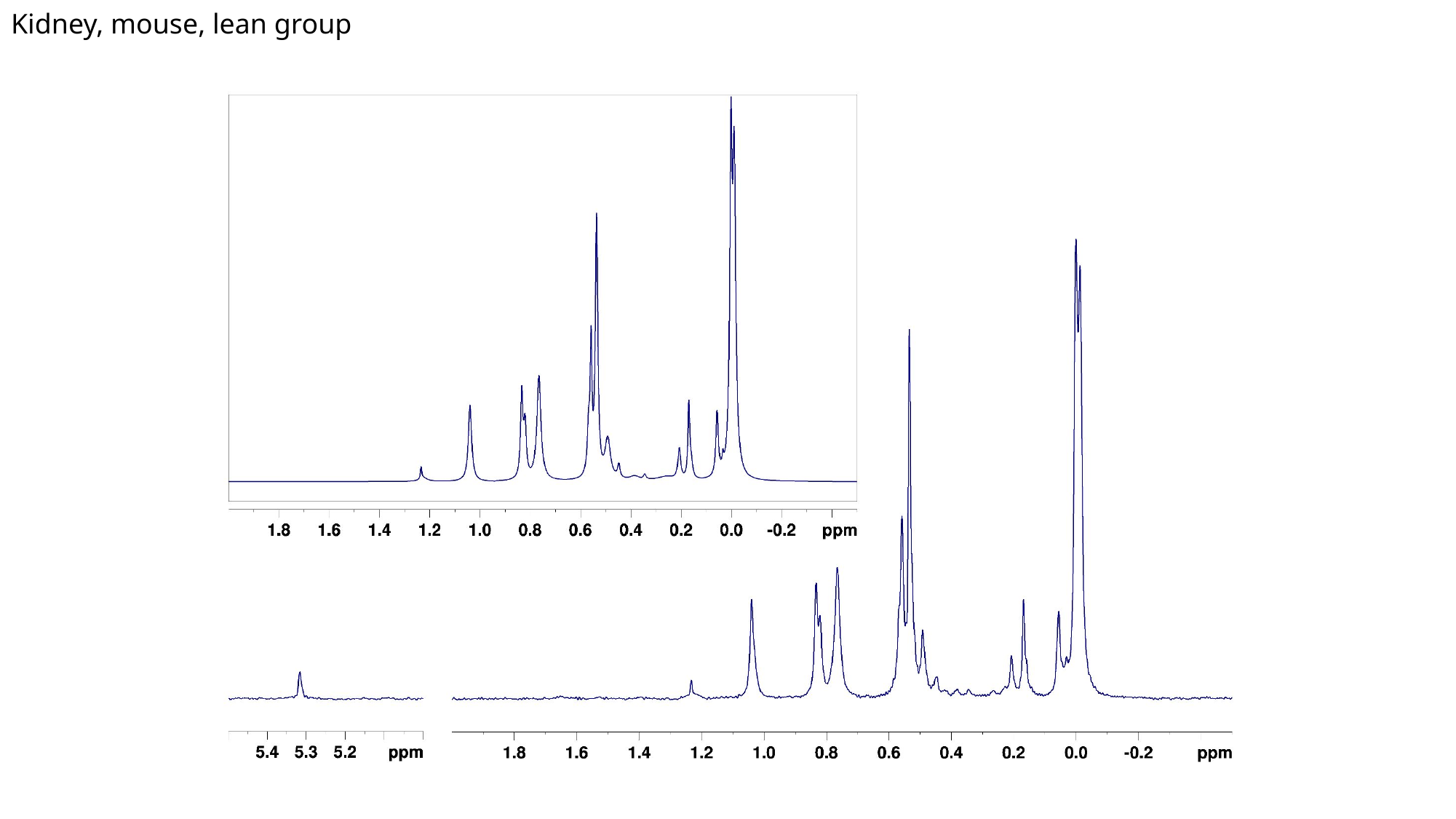

# Kidney, mouse, lean group

## Slide 3
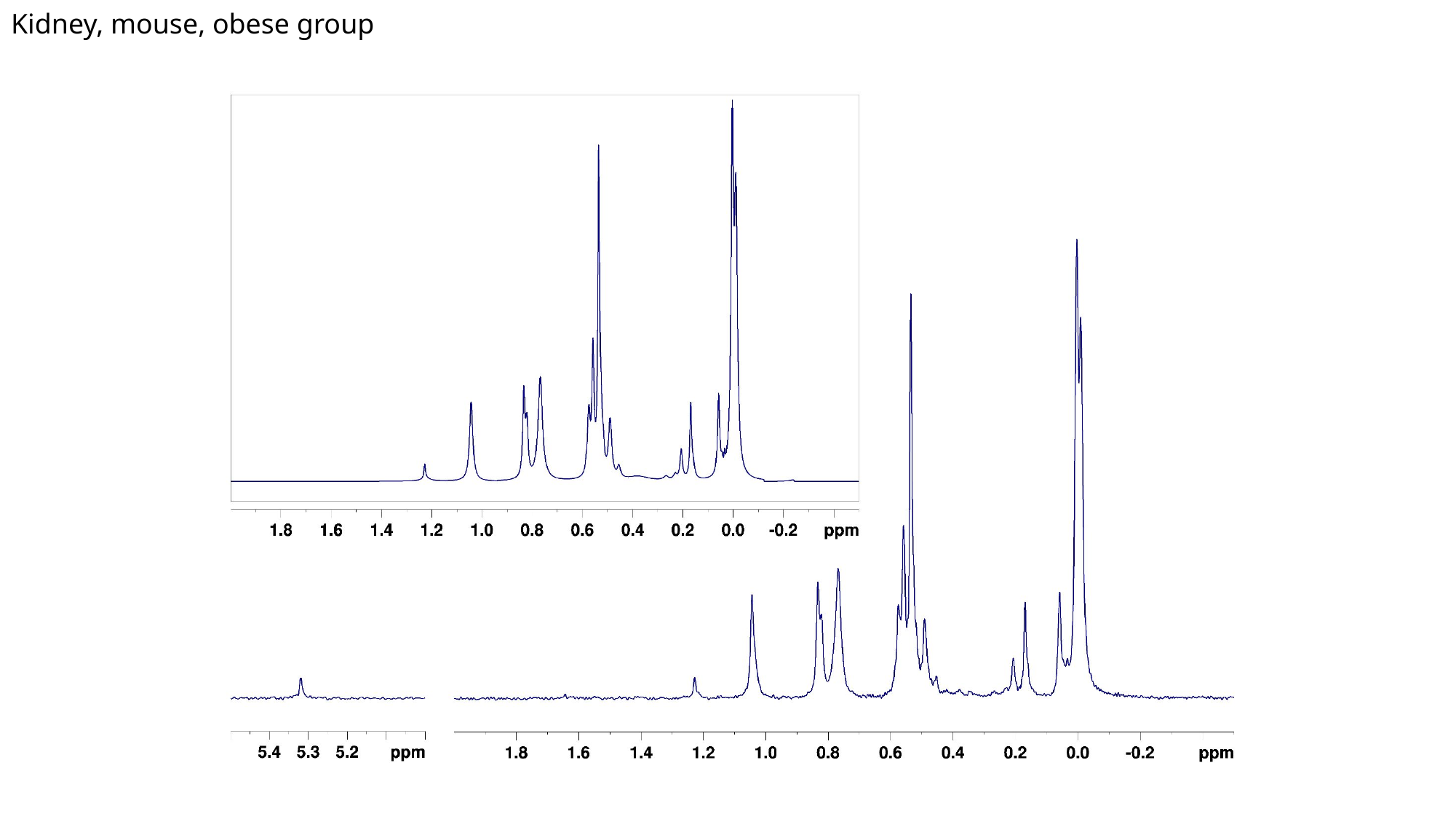

# Kidney, mouse, obese group

## Slide 4
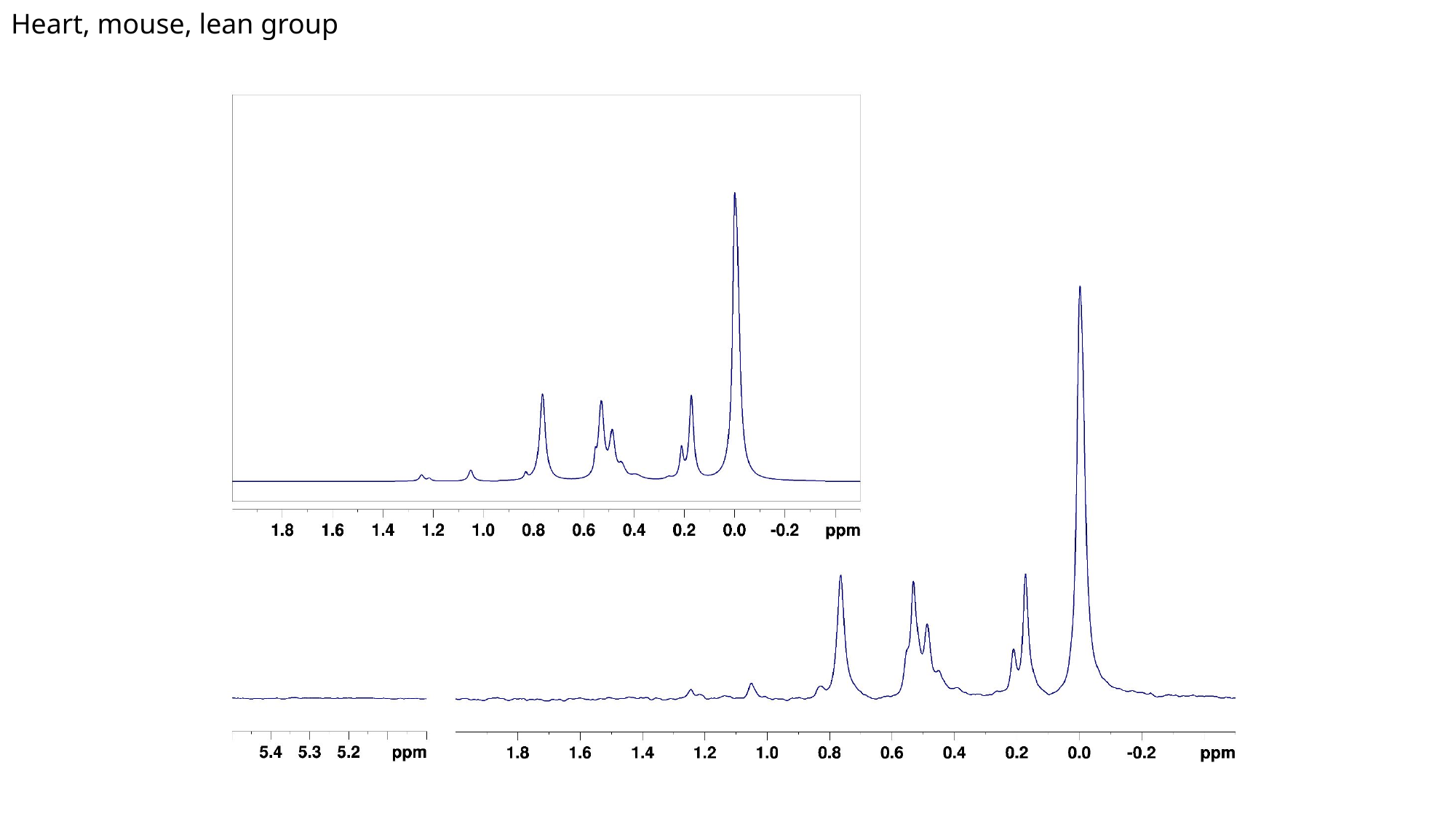

# Heart, mouse, lean group

## Slide 5
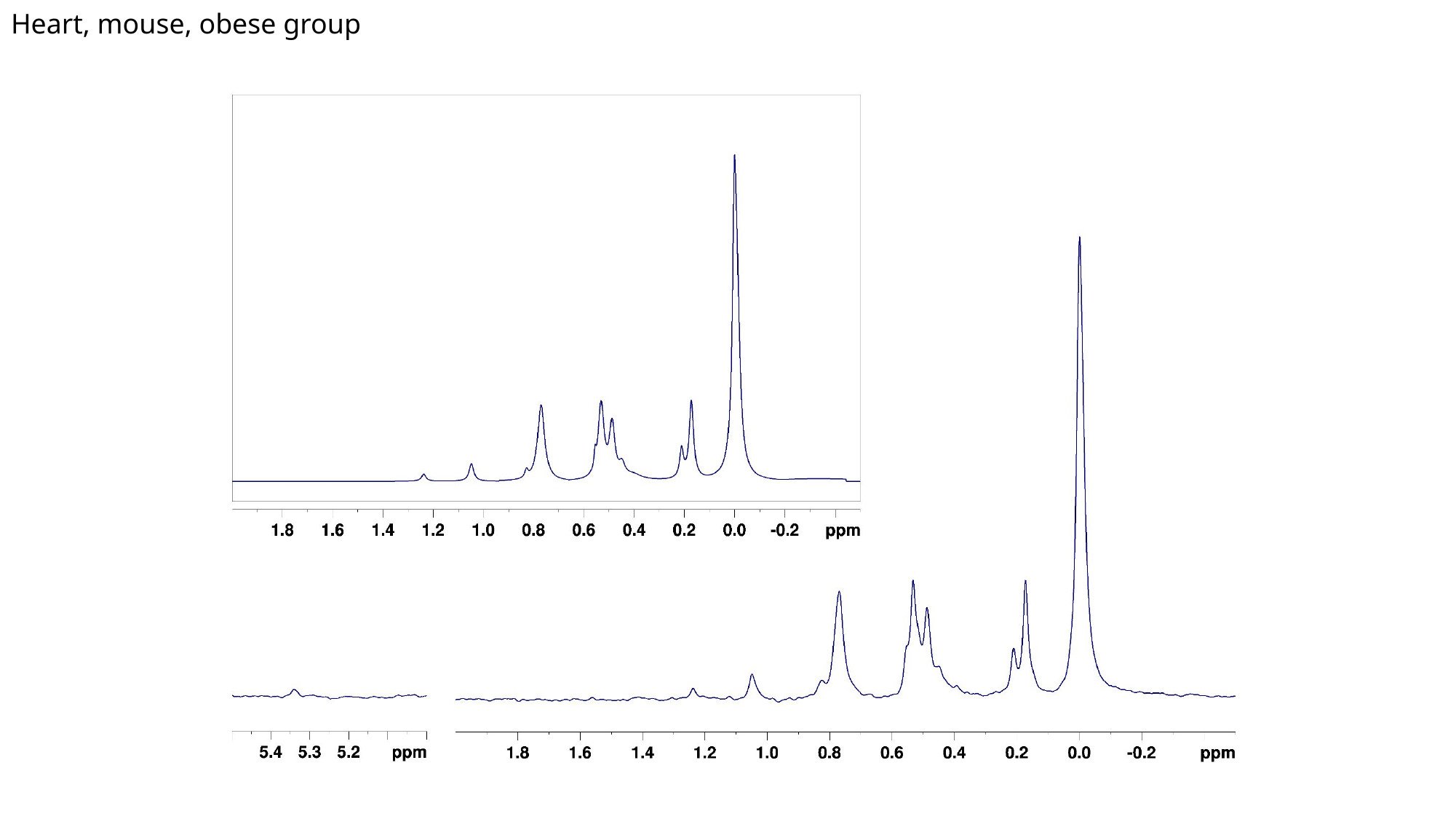

# Heart, mouse, obese group

## Slide 6
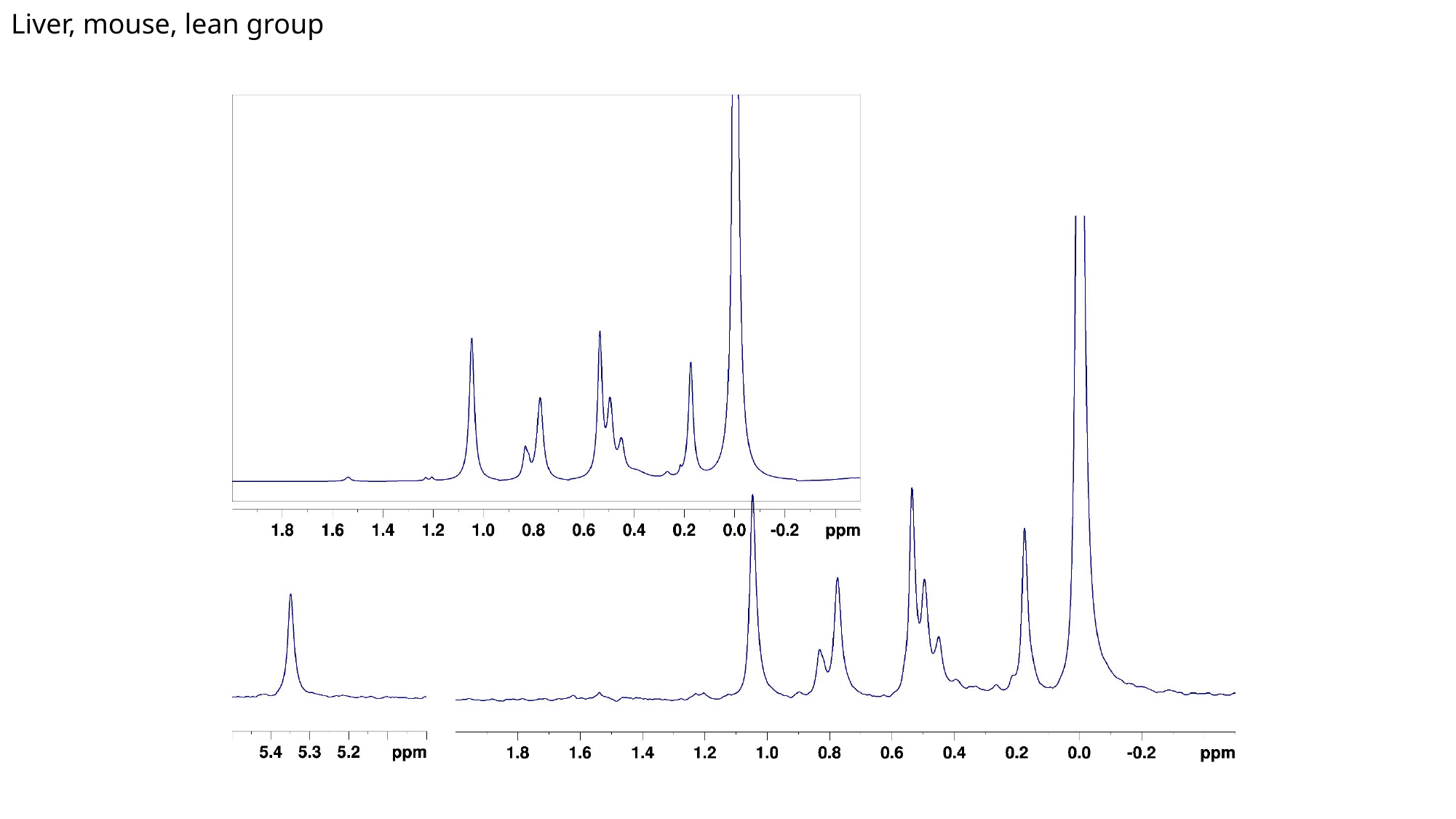

# Liver, mouse, lean group

## Slide 7
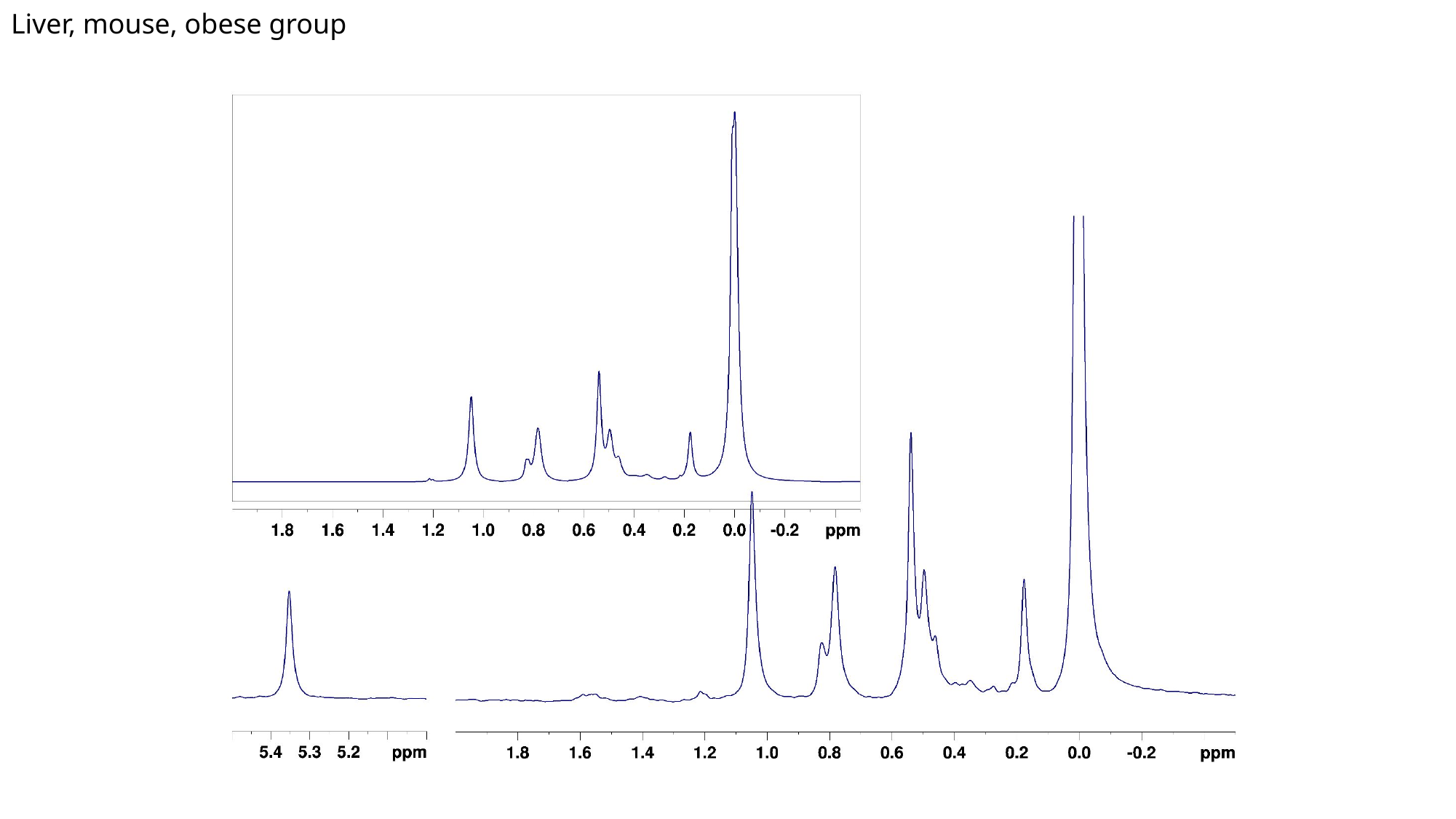

# Liver, mouse, obese group

## Slide 8
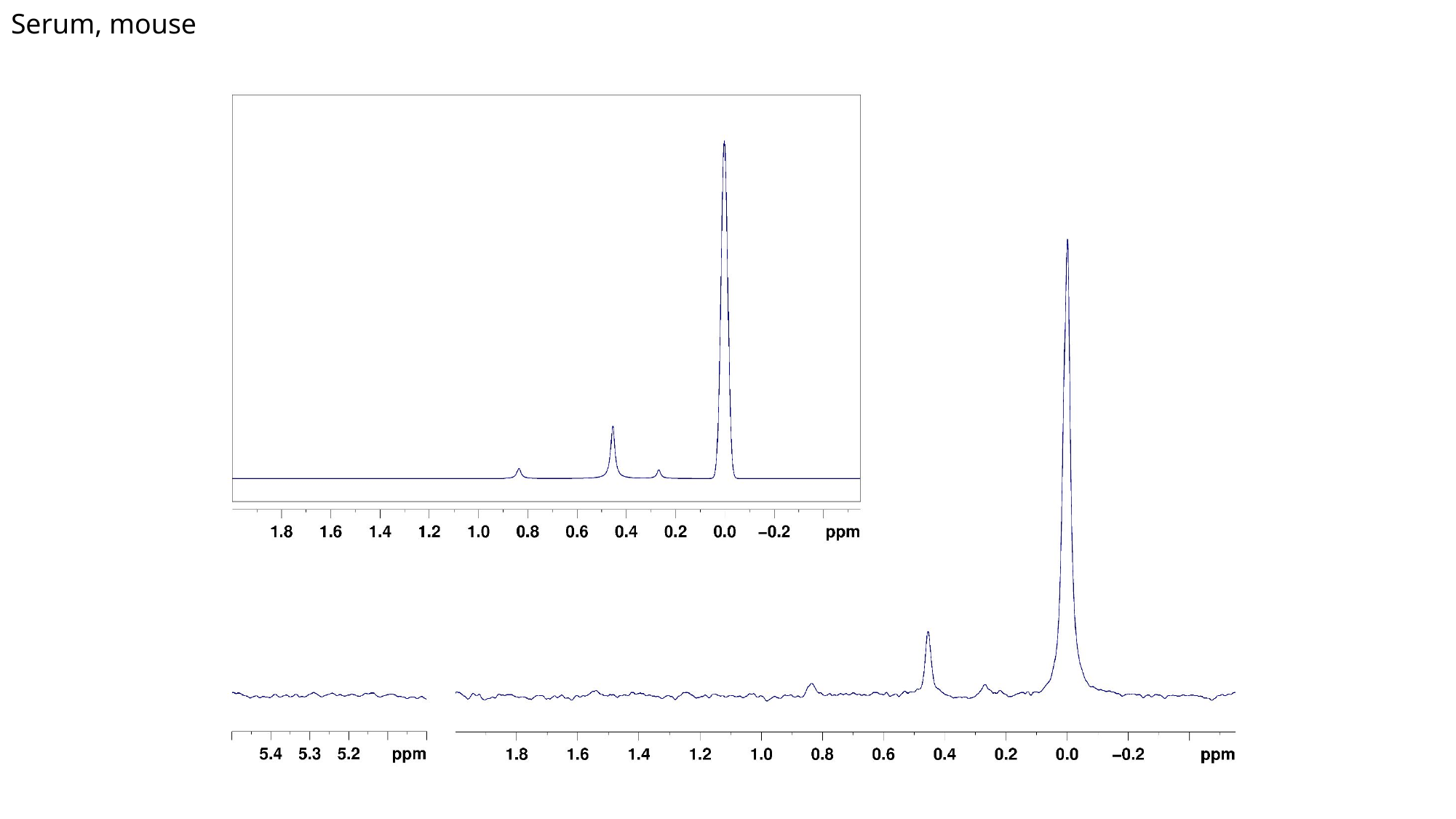

# Serum, mouse

## Slide 9
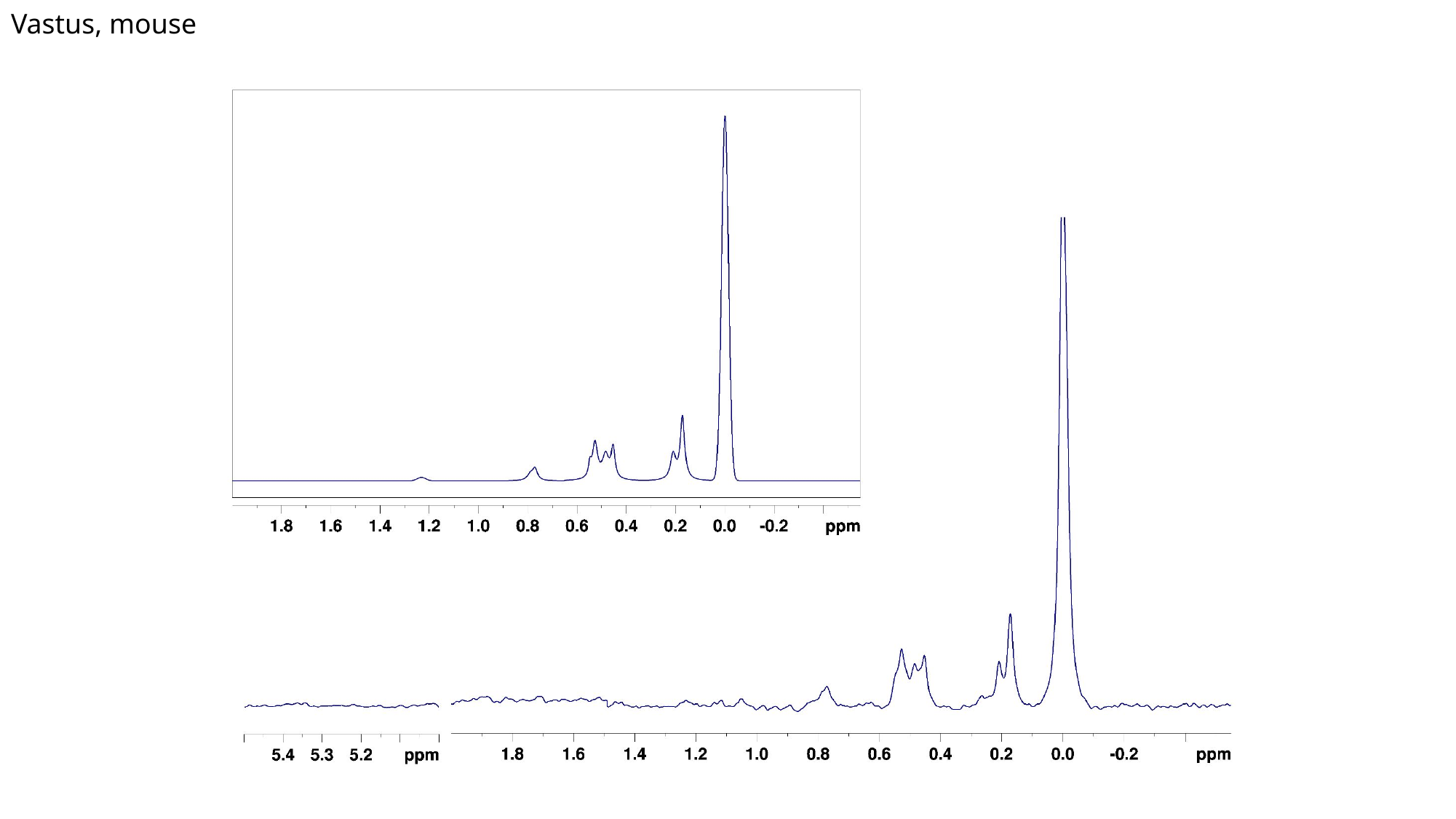

# Vastus, mouse

## Slide 10
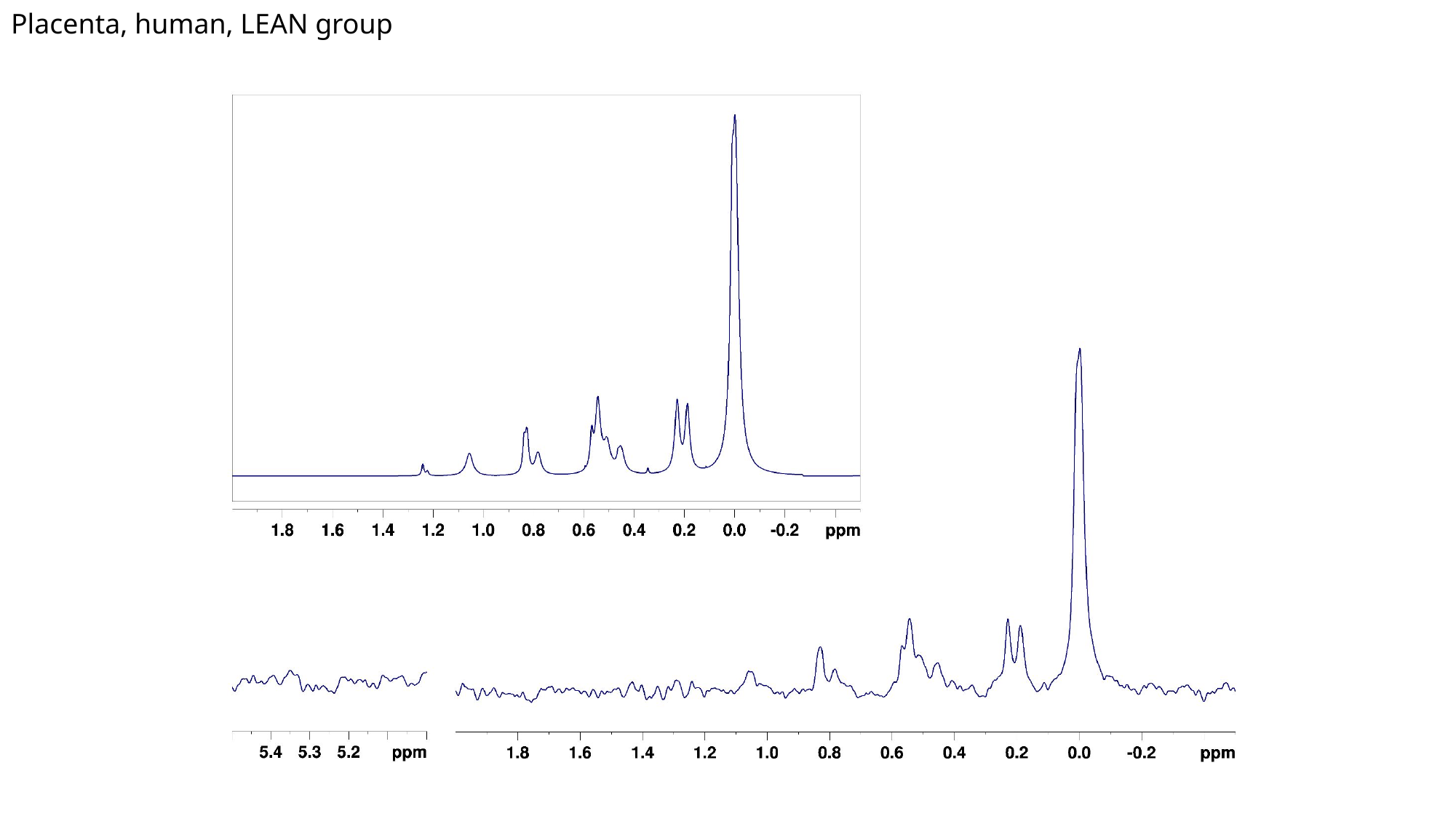

# Placenta, human, LEAN group

## Slide 11
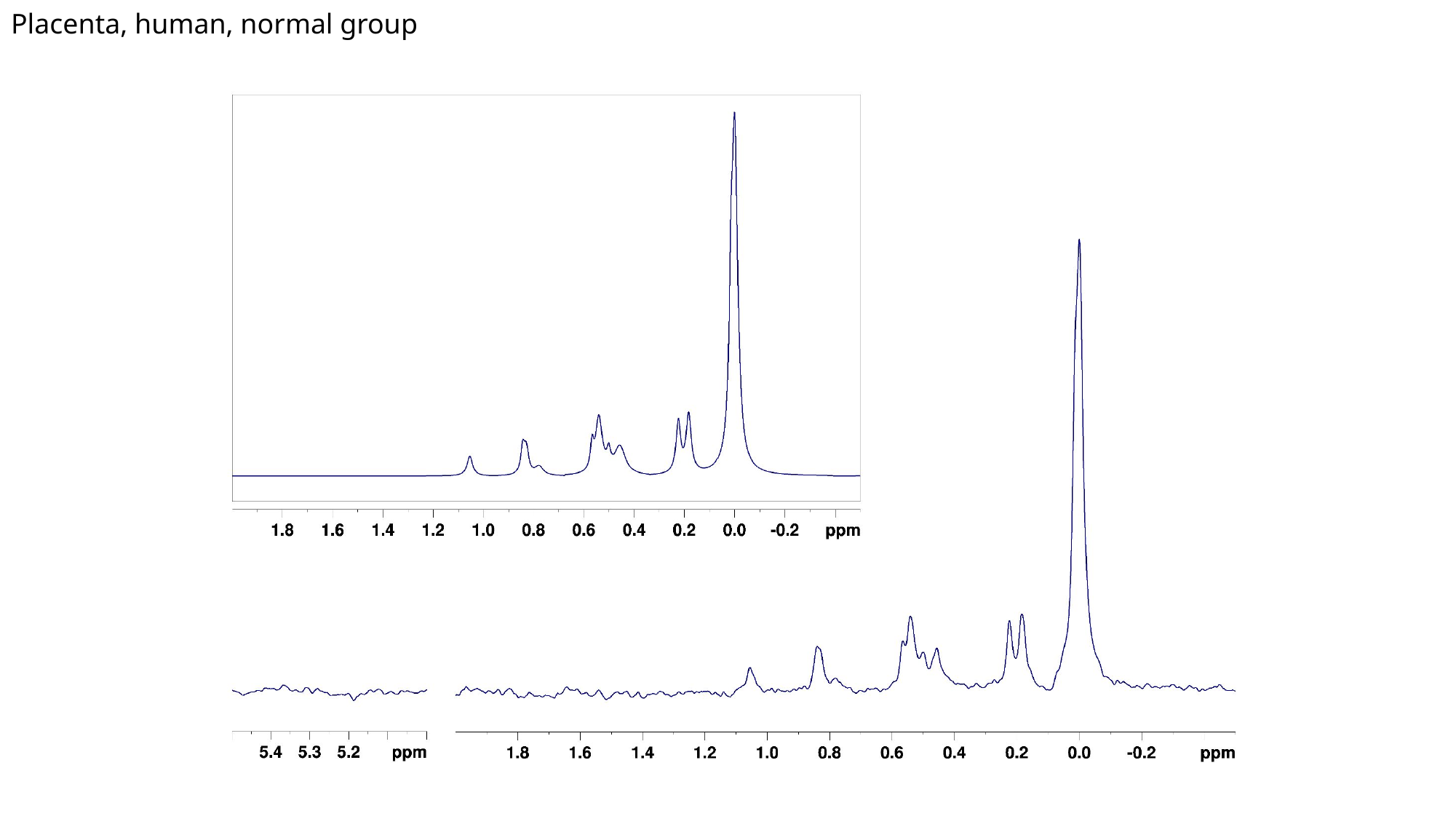

# Placenta, human, normal group

## Slide 12
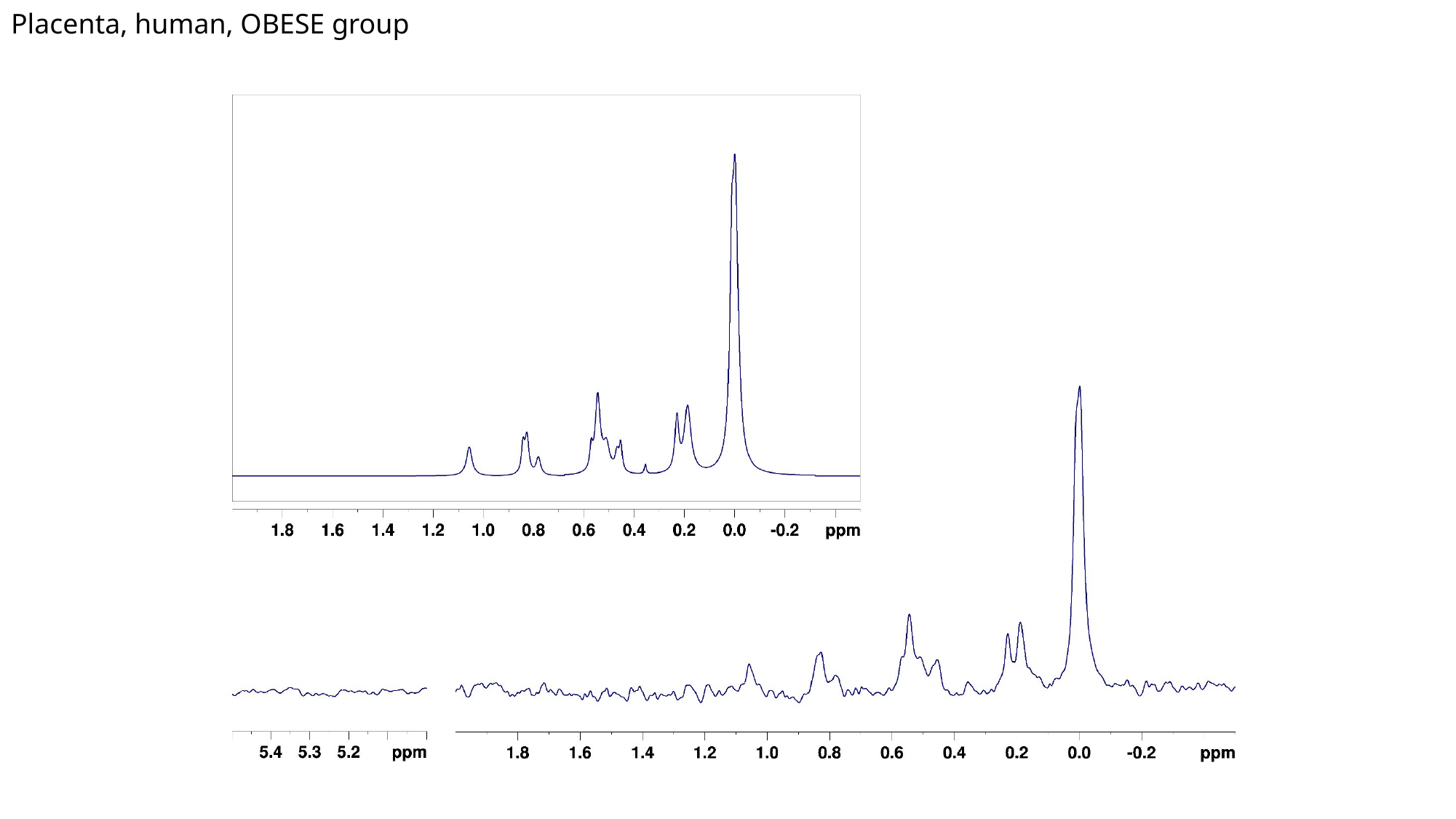

# Placenta, human, OBESE group

## Slide 13
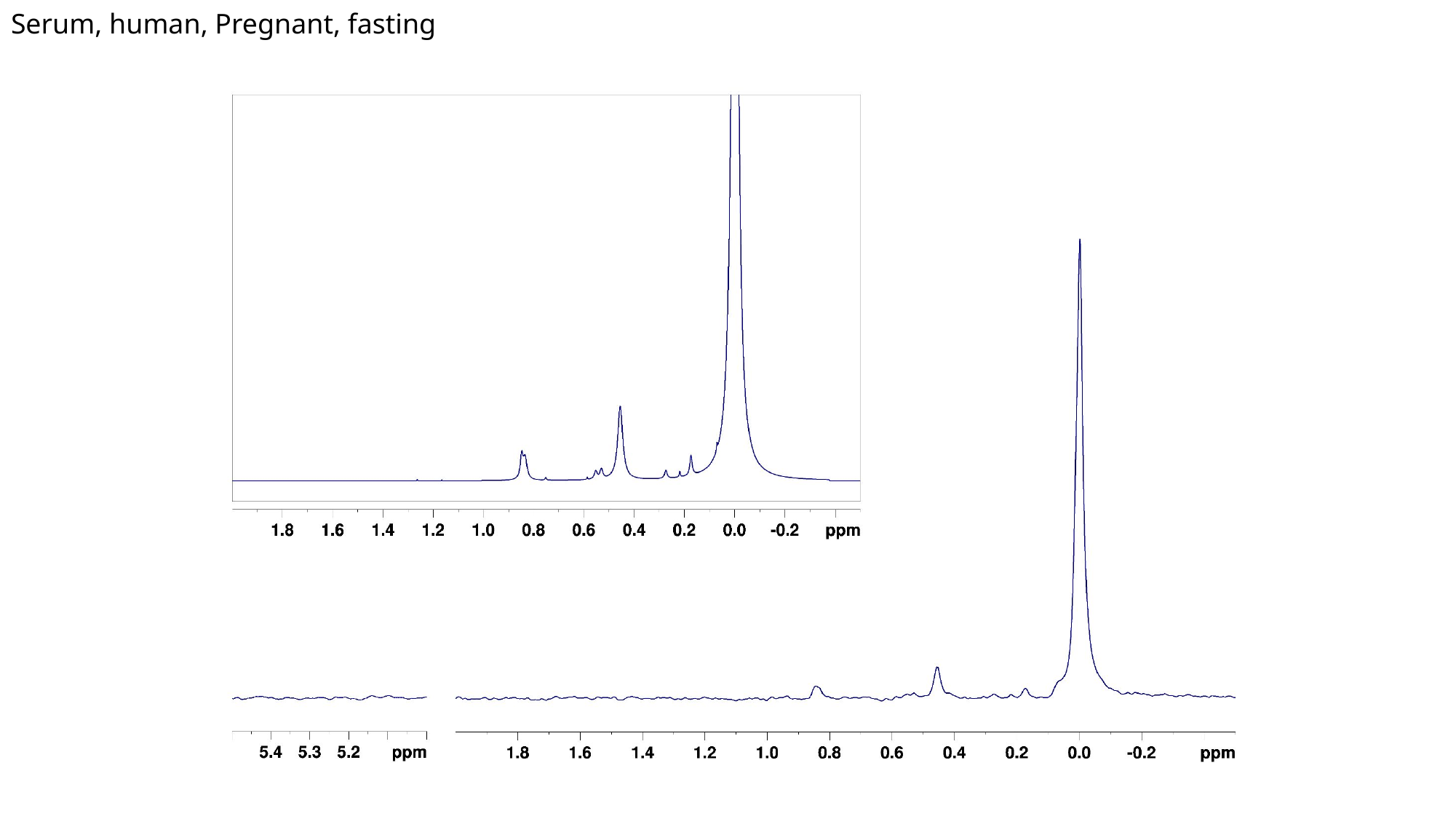

# Serum, human, Pregnant, fasting

## Slide 14
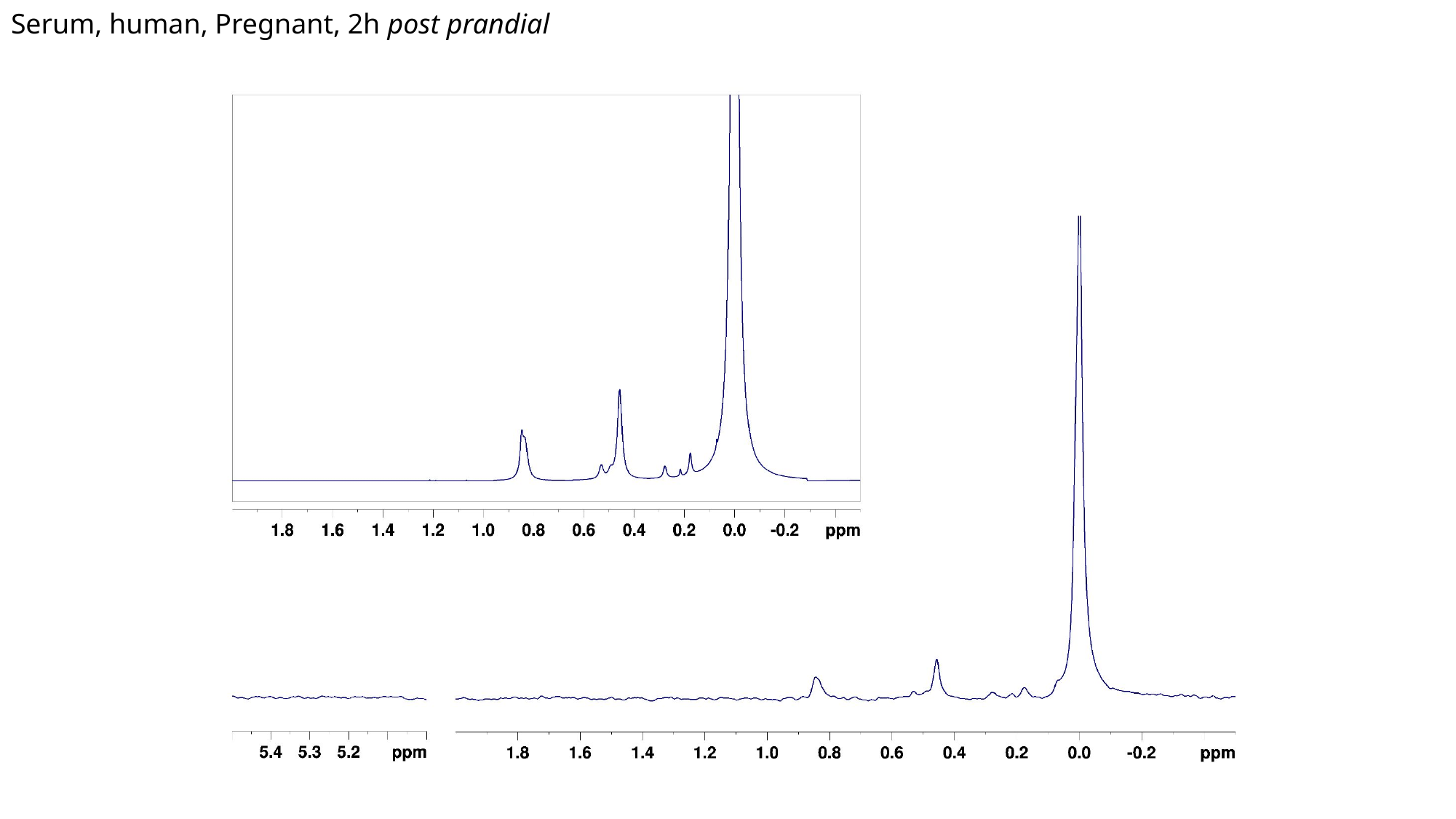

# Serum, human, Pregnant, 2h post prandial
