# Supplementary material for: A high-throughput platform for detailed lipidomic analysis of a range of mouse and human tissues
Source: Anal Bioanal Chem. 2020 Mar 7;412(12):2851–62. doi: 10.1007/s00216-020-02511-0 (PMC7196091; doi:10.1007/s00216-020-02511-0)
Supplement: Supplementary file 12 — (DOCX 1.48 mb) [file 216_2020_2511_MOESM12_ESM.docx]

**Analytical and Bioanalytical Chemistry**

**Electronic Supplementary Material**

**A high throughput platform for detailed lipidomic analysis of a range of mouse and human tissues**

Samuel Furse, Denise Fernandez-Twinn, Benjamin Jenkins, Claire L. Meek, Huw E. L. Williams, Gordon C. S. Smith, D. Stephen Charnock-Jones, Susan E. Ozanne, Albert Koulman

Additional files available under 10.1007/s00216-020-02511-0.

**Content**

**Supplementary Tables**

Table S1 – Participant information for human serum from pregnant and non-pregnant women

Table S2 – Participant information for Prediction Outcome Prediction (POP) Study

Table S3 – Preparation of tissue samples for extraction of the lipid fraction

Table S4 – Internal standards used

**Supplementary Figures**

Figure S1 – LC-MS used to determine the FA profile of phospholipid variables

Figure S2 – ^31^P NMR used to investigate the shift in phospholipid profile between kidneys of lean and obese mice

Figure S3 – ^31^P NMR used to investigate the shift in phospholipid profile between pregnant and non-pregnant samples

Figure S4 – 2D-^31^P NMR used to investigate the configuration of lipids associated with 1D ^31^P resonances

Figure S5 – Phospholipid variables unique to the livers and brains of either obese or lean individuals

Figure S6 – Activity of FADS2 through ratio of abundance of TG(50:3) with TG(50:2) from lean and obese mouse tissues

Figure S7 – Unsupervised multivariate analysis of the lipid profile of sera from pregnant and non-pregnant women

Figure S8 – Abundance of phospholipids that change significantly between pregnant and non-pregnant women at fasting at 2 h post prandial

Figure S9 – ^31^P NMR used to investigate the shift in phospholipid profile between lean and obese placenta samples

**Separate Files**

Representative ^31^P NMR spectra with deconvolutions (PPTX)

Signals files of mass spectrometry data for all tissue samples used in the present study (9 * XLSX)

Supplementary Information

Supplementary Tables

Table S1 Participant information for human serum samples from pregnant and non-pregnant women

|  | Non-pregnant | Pregnant* | Significance |
| --- | --- | --- | --- |
| n | 12 | 15 |  |
| Age (y) | 28.50 | 29.25 | 0.84 |
| BMI | 23.70 | 24.80 | 0.53 |
| BGC fasting (mM) | 5.12 | 4.13 | 4.78E-05 |
| BGC +2 h (mM) | 6.36 | 5.43 | 0.10 |
| HbA_1C_ | 34.20 | 30.73 | 0.04 |

*OGTT at 28 weeks’ gestation

BGC, blood glucose concentration; BMI, body mass index (based on weight (kg)/height (m) squared). +120 min refers to samples that were taken a number of minutes after ingestion of glucose (75 g) at fasting.

Table S2 Participant information for human placenta samples from Pregnancy Outcome Prediction Study

|  | Lean | Obese | Significance |
| --- | --- | --- | --- |
| n | 39 | 40 | - |
| Age (y) | 30.77 | 29.73 | 0.026 |
| BMI | 33.37 | 21.06 | 5.6 × 10^-25^ |
| Male offspring (%) | 58 | 28 | - |

Villious sections (30-40 mg) of placentae from vaginal singleton births.

Table S3. Preparation of tissue samples for extraction of the lipid fraction

| Tissue | Mass (mg) | GCTU (mL) | Freeze-thaw | Homogenisation | Storage | Further dilution | Vol. used in lipid extraction (µL) |
| --- | --- | --- | --- | --- | --- | --- | --- |
| Human serum | n/a | 0 | 0 | - | -80 °C | n/a | 20 |
| Mouse serum | n/a | 0 | 0 | - | -80 °C | n/a | 20 |
| Mouse adipose | ~250 | 2 | n/a | TissueRuptor II | -80 °C | Methanol (400 µL) and  TBME (100 µL) | 8 |
| Mouse brain | ~450 | 2 | n/a | TissueRuptor II | -80 °C | n/a | 30 |
| Mouse kidney | ~200 | 1 | n/a | TissueRuptor II | -80 °C | n/a | 50 |
| Mouse liver | ~400 | 2 | n/a | TissueRuptor II | -80 °C | n/a | 10 |
| Mouse heart | ~100 | 1 | 1× | Pestle & mortar* | -80 °C | n/a | 50 |
| Mouse vastus muscle | ~150 | 0.6 | 3×, 1× after homog.** | TissueRuptor II^†^ | -80 °C | n/a | 50 |
| Human placenta (portion) | 30-40 | 0.7 | n/a | TissueRuptor II | -80 °C | n/a | 50 |

*Before being dispersed in GCTU, samples ground to a powder at -78 °C. **Samples were checked for the presence of further solid material after freeze-thawing, those with solid material were homogenized again. ^†^ Samples were centrifuged briefly after homogenization, before being frozen (<20 s, up to 5k × *g*).

Table S4 List of internal standards used for lipid profiling in the present study

| **Lipid Class** | **Isoform** |
| --- | --- |
| Cholesteryl ester | CE(18:0-d_6_ ) |
| Ceramide | C16-d_31_ Ceramide |
| Fatty acid | C15:0-d_29_ FA |
| Fatty acid | C17:0-d_33_ FA |
| Fatty acid | C20:0-d_39_ FA |
| lyso-Phosphatidylcholine | lysoPC(C14:0)-d_42_ |
| Phosphatidic acid | PA(C16:0-d_31_/C18:1) Na^+^ salt |
| Phosphatidylcholine | PC(C16:0-d_31_/C18:1) |
| Phosphatidylethanolamine | PE(C16:0-d_31_/C18:1) |
| Phosphatidylglycerol | PG(C16:0-d_31_/C18:1) Na^+^ salt |
| Phosphatidylinositol | PI(C16:0-d_31_/C18:1) NH_4_^+^ salt |
| Phosphatidylserine | PS(C16:0-d_62_) Na^+^ salt |
| Sphingomyelin | SM(C16:0-d_31_) |
| Triglyceride | TG(45:0-d_29_) |
| Triglyceride | TG(48:0-d_31_) |
| Triglyceride | TG(54:0-d_35_) |

Supplementary Figures

LC‑MS used to determine the FA profile of phospholipid variables


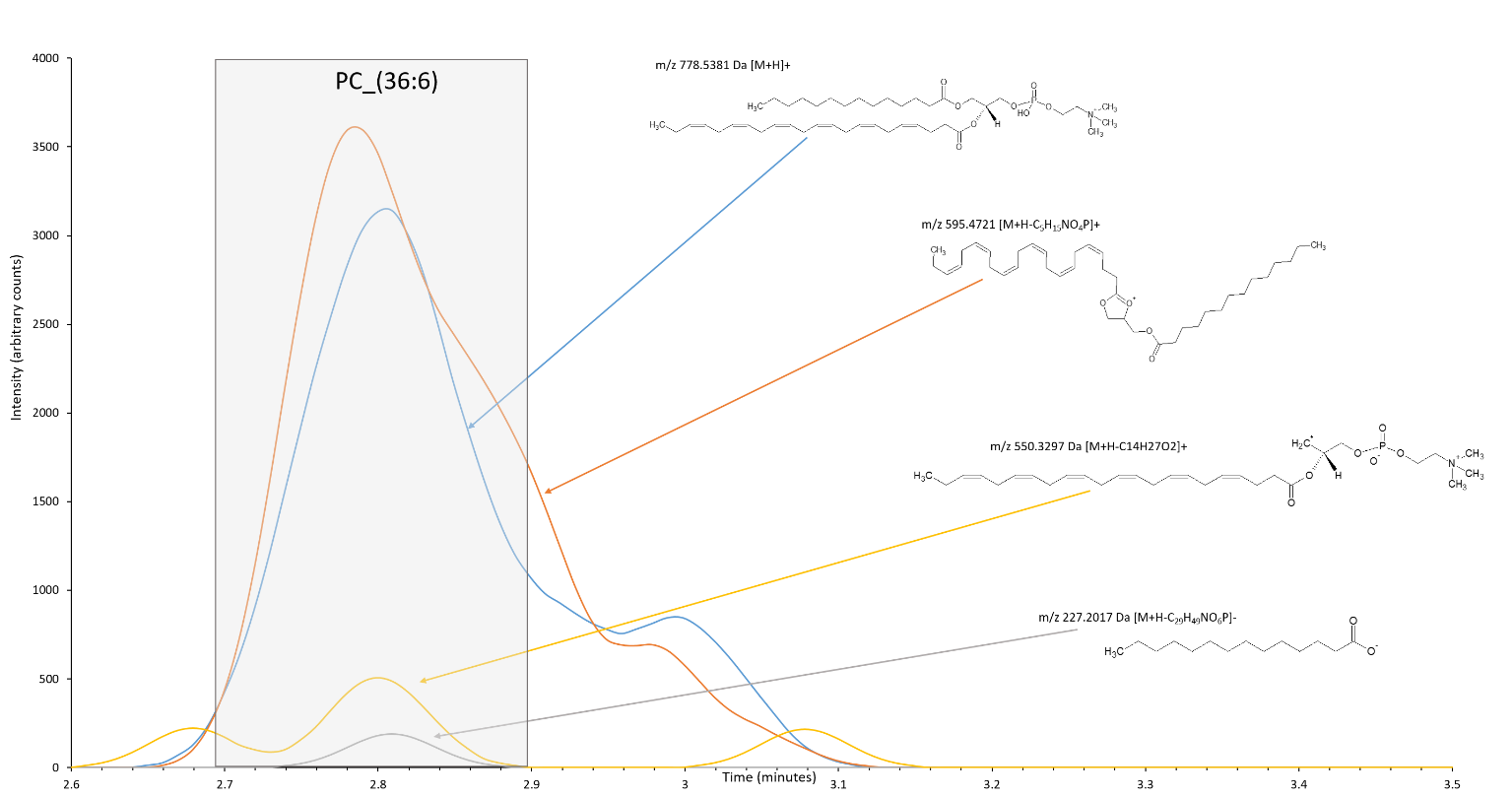


Fig. S1. Analysis of PC(36:6) using LC-MS in both positive and negative ionization modes with collision induced dissociation (CID): positive/positive CID switching and negative/negative CID switching. Fragments observed across both modes are shown. CID, Collision-induced dissociation/in-source fragmentation.

^31^P NMR used to investigate the shift in phospholipid profile between kidneys of lean and obese mice


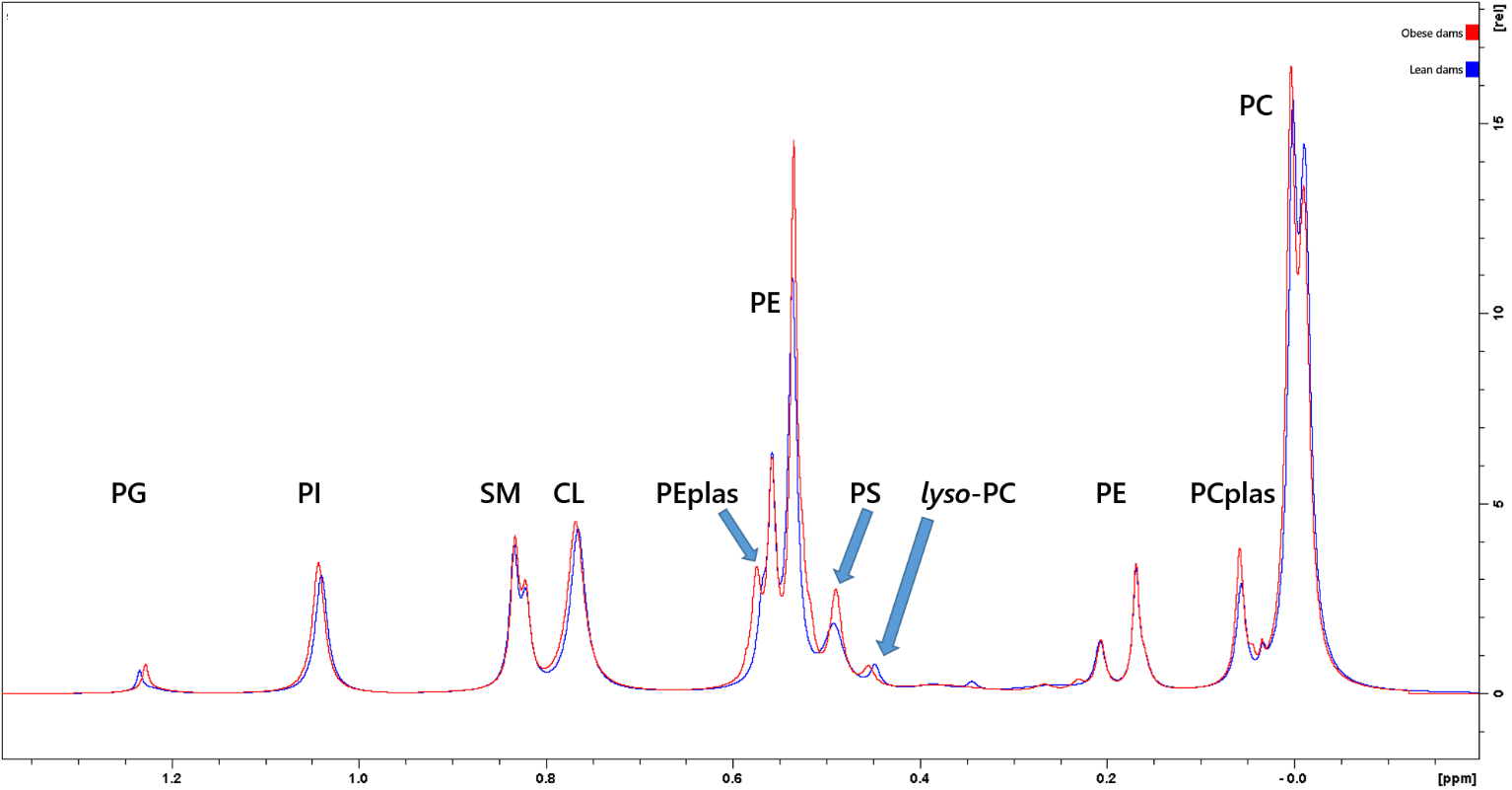


Fig. S2. Stacked, deconvoluted, 1D ^31^P NMR spectra from pooled mouse kidney samples collected from lean and obese post-weaning dams. Phosphatidylethanolamine signals appear at 0·56, 0·53, 0·21, 0·17 ppm (See *Fig. S4*). CL, cardiolipin; *lyso-*PC, *lyso-*phosphatidylcholine; PC, phosphatidylcholine; PCplas, plasamalogen-phosphatidylcholine; PE, phosphatidylethanolamine; PEplas, plasmalogen-phosphatidylethanolamine; PG, Phosphatidylglycerol; PI, phosphatidylinositol; PS, phosphatidylserine; SM, sphingomyelin.

^31^P NMR used to investigate the shift in phospholipid profile between pregnant and non-pregnant samples


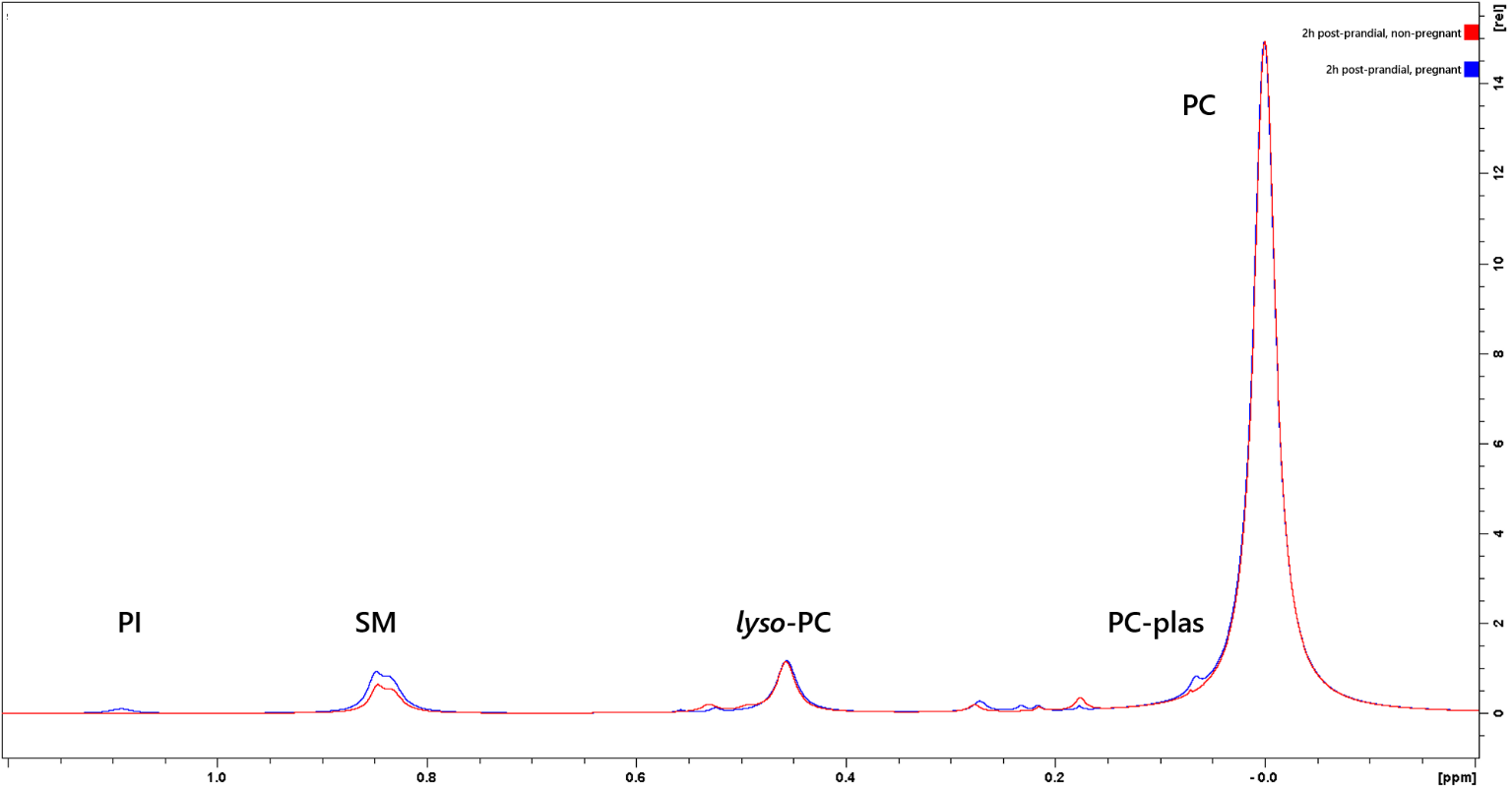


Fig. S3. Stacked, deconvoluted, 1D ^31^P NMR spectra from pooled human serum samples collected from pregnant and non-pregnant women during an oral glucose tolerance test. Phosphatidylethanolamine signals appear at 0·55, 0·28, 0·18 ppm (See *Fig. S4*). *lyso-*PC, *lyso-*phosphatidylcholine; PC, phosphatidylcholine; PCplas, plasamalogen-phosphatidylcholine; PI, phosphatidylinositol; SM, sphingomyelin.

2D-^31^P NMR used to investigate the configuration of lipids associated with 1D ^31^P resonances


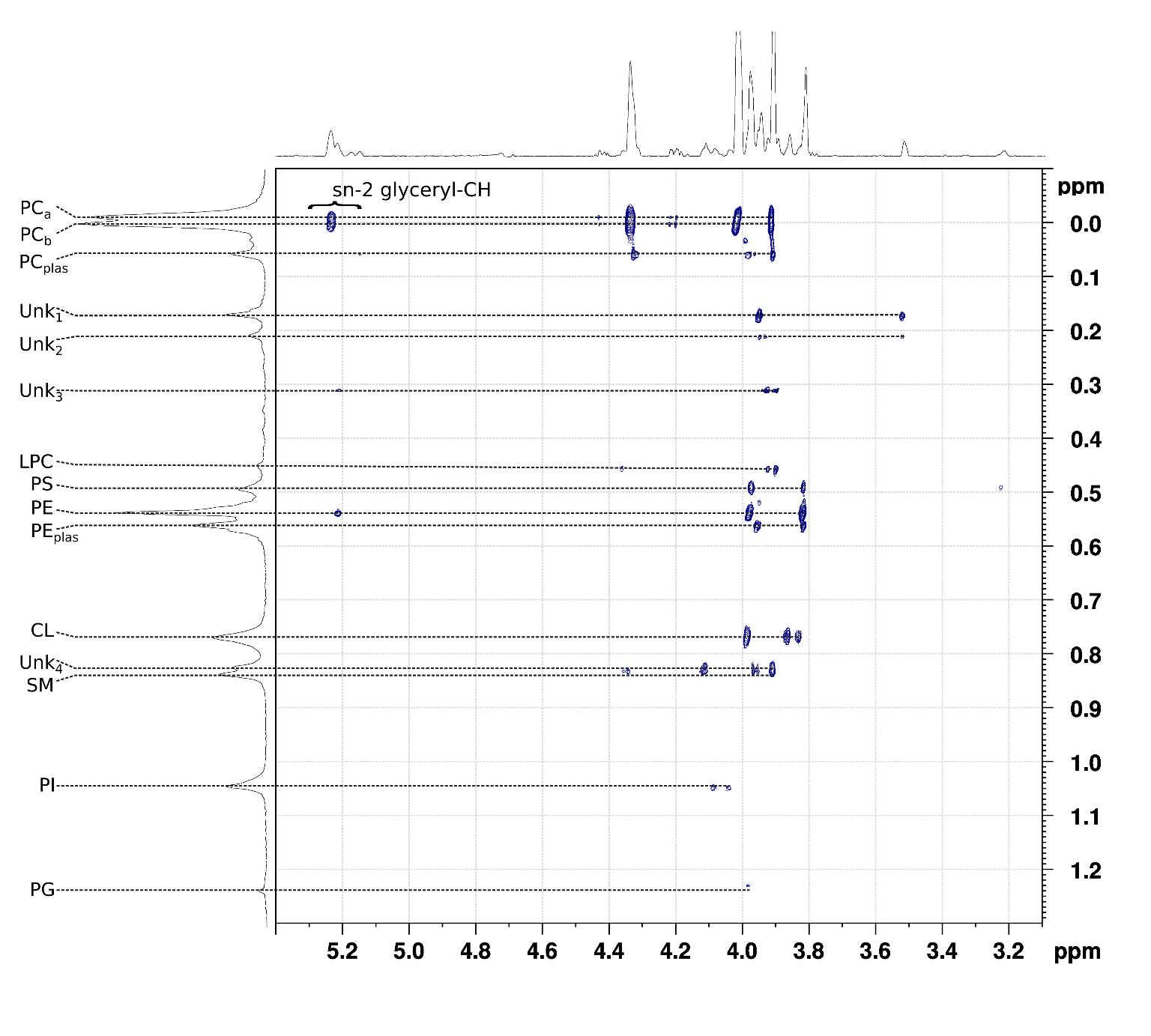


Fig. S4. ^31^P-HSQC spectrum of kidney from obese post-weaning mouse dams. This suggests that phosphatidylethanolamine signals appear at 0·56, 0·53, 0·21, 0·17 ppm (Unk_1_, Unk_2_), that PCplas has a shift of 0·05 ppm and that the resonances for SM and PC can be split as well as those for PG and PE. CL, cardiolipin; LPC, *lyso-*phosphatidylcholine; PC, phosphatidylcholine; PCplas, plasamalogen-phosphatidylcholine; PE, phosphatidylethanolamine; PEplas, plasmalogen-phosphatidylethanolamine; PG, Phosphatidylglycerol; PI, phosphatidylinositol; PS, phosphatidylserine; SM, sphingomyelin.

Phospholipid class abundance in the livers and brains of obese and lean mice

A

B

Fig. S5. The average relative abundance of phospholipid classes in the livers (top) and brains (bottom) of lean and obese groups of mice. Measured by positive ionisation mode mass spectrometry. Error bars show standard deviation. Cer, ceramide; LPC, *lyso-*phosphatidylcholine; PC, phosphatidylcholine; PC-O, plasamalogen-phosphatidylcholine; PE, phosphatidylethanolamine; PE-O, plasmalogen-phosphatidylethanolamine; PG, Phosphatidylglycerol; PI, phosphatidylinositol; PS, phosphatidylserine; SM, sphingomyelin; TG, triglyceride.

Activity of FADS2 through ratio of abundance of TG(50:3) with TG(50:2) from lean and obese mouse tissues

Fig. S6. The ratio of TG(50:03)/ TG(50:02) in tissues from lean and obese mice. Values calculated from the mean relative abundance of the TG(50:03) divided by the mean relative abundance of TG(50:02) (see signals sheets for calculations). This ratio implies the activity of fatty acid desaturase 2 (FADS2). TG, triglyceride.

Unsupervised multivariate analysis of the lipid profile of sera from pregnant and non-pregnant women


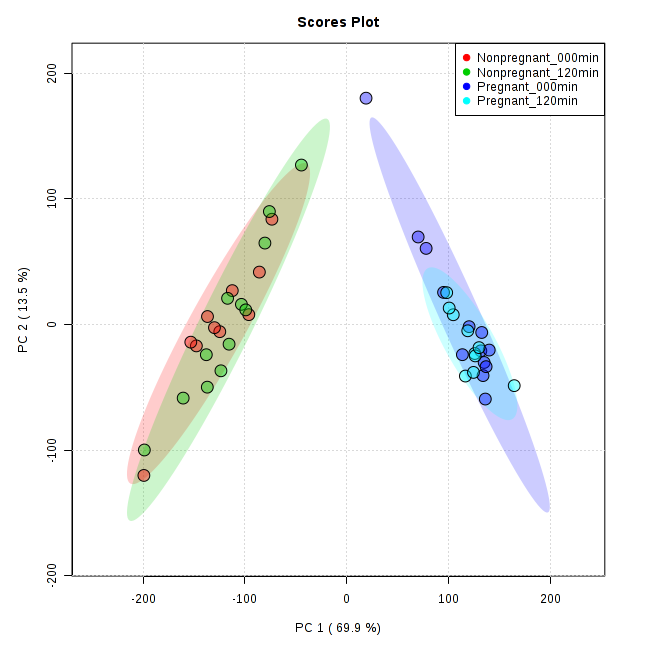


Fig. S7. Principal component analysis of the lipid profiles of serum samples from pregnant and non-pregnant women both at fasting (000 min) and 120 min *post prandial* (after ingestion of glucose, 75 g).

Abundance of phospholipids that change significantly between pregnant and non-pregnant women at fasting at 2 h post prandial

Fig. S8. Abundance of lipids in serum samples from pregnant and non-pregnant women both at fasting (0 h) and 2 h after ingestion of glucose (75 g), measured in negative ionization mode.

^31^P NMR used to investigate the shift in phospholipid profile between lean and obese placenta samples


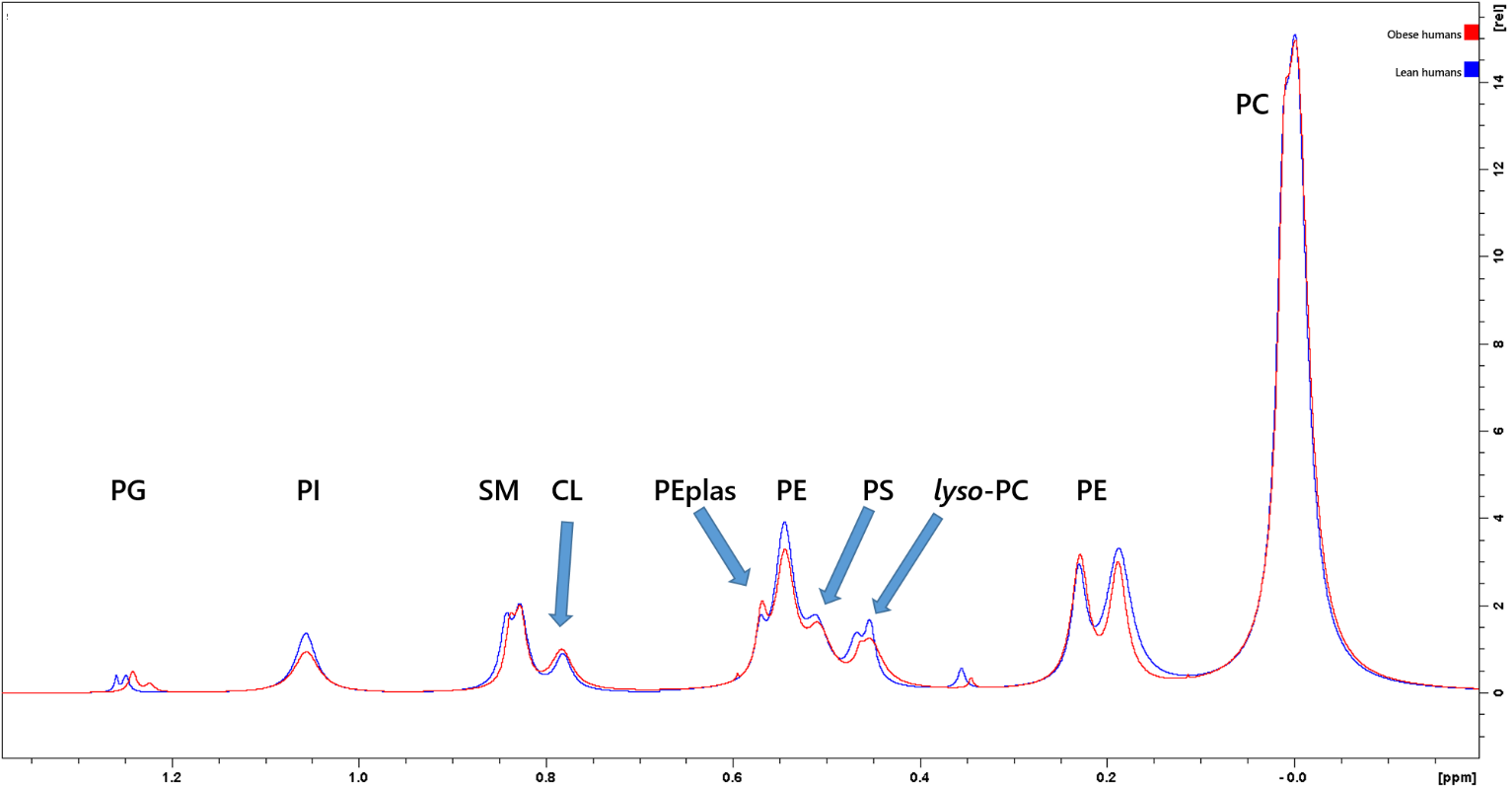


Fig. S9. Stacked, deconvoluted, 1D ^31^P NMR spectra from pooled human placentae samples collected from lean and obese vaginal singleton male births. Phosphatidylethanolamine signals appear at 0·54, 0·24, 0·18 ppm, See *Fig. S4*. CL, cardiolipin; LPC, *lyso-*phosphatidylcholine; PC, phosphatidylcholine; PC-O, plasamalogen-phosphatidylcholine; PE, phosphatidylethanolamine; PE-O, plasmalogen-phosphatidylethanolamine; PG, Phosphatidylglycerol; PI, phosphatidylinositol; PS, phosphatidylserine; SM, sphingomyelin.
